# Computational and Experimental Realization of Metal-Ion-Doped Orthorhombic Sn_3_O_4_ for Visible-Light-Active Photocatalysis

**DOI:** 10.1021/jacs.5c15962

**Published:** 2026-02-03

**Authors:** Sho Uchida, Yuta Sekine, Yohei Cho, Akira Yamaguchi, Toyokazu Tanabe, Kenji Yamaguchi, Masahiro Miyauchi

**Affiliations:** † Department of Materials Science and Engineering, School of Materials and Chemical Technology, 13290 Institute of Science Tokyo, Tokyo 152-8552, Japan; ‡ Department of Materials Science and Engineering, 13314 National Defense Academy, Kanagawa 239-0811, Japan; § Innovation Center, 88377 Mitsubishi Materials Corporation, Ibaraki 311-0102, Japan

## Abstract

The orthorhombic tri-tin tetraoxide (Sn_3_O_4_) is a newly discovered polymorph and has attracted great attention due to its visible-light absorption capability. To improve performance and broaden the material space based on orthorhombic Sn_3_O_4_, impurity doping represents a promising approach. In this study, we predict stable cation-doped orthorhombic Sn_3_O_4_ crystals using machine learning interatomic potential (MLIP) calculations. Several candidate cations such as boron (B), aluminum (Al), strontium (Sr), and yttrium (Y) have been predicted as stable dopants in orthorhombic Sn_3_O_4_ with low Gibbs energies of formation. Based on this prediction, we synthesized cation-doped Sn_3_O_4_ powder samples using a hydrothermal method. We confirmed that the cations predicted to be stable by the MLIP could be synthesized into the orthorhombic powder phase. Among the samples, the Al-doped Sn_3_O_4_ powder exhibited superior photocatalytic hydrogen production activity under visible light. Furthermore, we fabricated thin films of Al-doped Sn_3_O_4_ and optimized the doping amount of Al to achieve high photocatalytic activity. The 5% Al-doped Sn_3_O_4_ exhibited the highest activity owing to its high crystallinity and optimal morphology for better separation of photogenerated carriers. The Al-doped orthorhombic Sn_3_O_4_ is promising for application as a visible light-active photocatalyst.

## Graphical abstract

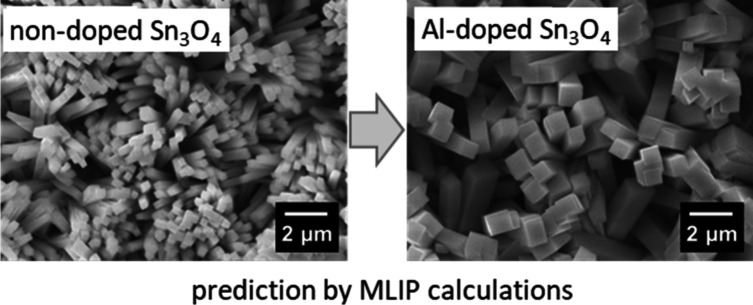

## Introduction

Tin oxides are robust and nontoxic, making them attractive candidates for stable photocatalysts for environmental purification and energy production.^1–3^ Most of the reported tin oxides are in SnO or SnO_2_ forms, having divalent (Sn^2+^) or tetravalent (Sn^4+^) oxidation states. In addition to these tin oxides, mixed-valence Sn_3_O_4_, i.e., (Sn^2+^)_2_(Sn^4+^)­O_4_, has attracted significant attention because the coexistence of Sn^2+^ and Sn^4+^ orbitals contributes to a valence band upshift, enabling visible-light absorption.^4,5^ Although the crystal structure of the reported Sn_3_O_4_ was limited to the monoclinic^6–8^ or triclinic phase,^9^ its photocatalytic properties have been widely studied.^10–12^ For example, the internal quantum efficiencies of the monoclinic Sn_3_O_4_ powder and film without any cocatalyst modification were only 1.57 × 10^–4^ and 1.58 × 10^–4^%, respectively.^13^ Many researchers have modified them by impurity ion doping^14–19^ and/or combined them with other materials^20–26^ to improve their visible-light activities. Very recently, our group established the synthesis of a new polymorph of Sn_3_O_4_, i.e., the orthorhombic Sn_3_O_4_, by a facile hydrothermal process other than the monoclinic and triclinic phases.^27^ The bandgap of orthorhombic Sn_3_O_4_ was narrower than that of monoclinic Sn_3_O_4_, and the photocatalytic activity of orthorhombic Sn_3_O_4_ was higher than that of monoclinic Sn_3_O_4_.^13^

Although the synthesis of orthorhombic Sn_3_O_4_ has been established recently, attempts at impurity doping of this crystal have not yet been investigated. Impurity doping is regarded as a promising strategy in photocatalysis, as it can introduce reaction centers, modify crystal structures, and tune defect states or absorption properties.^28–31^ These effects have been investigated in many established materials, largely through empirical trial-and-error approaches. For newly discovered semiconductors, however, it is essential to first assess whether stable doping can be achieved, particularly in metastable systems such as orthorhombic Sn_3_O_4_. Therefore, in this study we initially employed a machine-learning interatomic potential (MLIP)^32^ to evaluate the stability of substitutional dopants across the periodic table. This approach enabled us to narrow down the candidate elements without the extensive computational cost required for large-scale density functional theory (DFT) calculations. Based on this theoretical calculation, we identified several candidate cations that can be stably doped into orthorhombic Sn_3_O_4_. We then attempted to synthesize these cation-doped Sn_3_O_4_ samples using a hydrothermal method experimentally. We confirmed that the cations predicted by the calculations to be stable, could indeed be incorporated into the orthorhombic powder phase. We also compared the photocatalytic hydrogen production activities of various cation-doped Sn_3_O_4_ powder under visible light irradiation and found that the aluminum (Al)-doped Sn_3_O_4_ with the orthorhombic phase exhibited superior photocatalytic activity to the other doped Sn_3_O_4_ or to the nondoped orthorhombic Sn_3_O_4_. Furthermore, we developed thin films of Al-doped Sn_3_O_4_ with an optimized Al doping density for high photocatalytic activity. We comprehensively investigated the physicochemical properties and photoelectrochemical and photocatalytic activities of Al-doped Sn_3_O_4_ films and discussed the mechanism of their enhanced photocatalytic activities.

## Resutls and Discussion

### Computational Screening of Dopant into Orthorhombic Sn_3_O_4_

To screen stable dopants in orthorhombic Sn_3_O_4_, electrically neutral cation-doped orthorhombic Sn_3_O_4_ models generated via direct substitution without charge compensation were evaluated using preferred potential (PFP),^32^ selected among the universal MLIPs^33^ to estimate their Gibbs formation energies. The training data for MLIP were obtained from DFT calculations without Hubbard *U* corrections; the detailed calculation method is described in the experimental details in the Supporting Information. Panels a and b of Figure 1 show the crystal models for doped Sn_3_O_4_ into the Sn^2+^ site at the Wyckoff positions of 4g or 4h, and into the Sn^4+^ site at the 4f position, respectively. Each unit cell had eight Sn^2+^ ions and four Sn^4+^ ions (a total of 12 tin ions) with a 2 × 2 × 2 supercell. The foreign cation was substituted at the Sn^2+^ or Sn^4+^ sites of Sn_3_O_4_ at a ratio of 1 atom per 96 (approximately 1 at. %). The effect of finite temperature (500 K) was evaluated within the range of the quasi-harmonic approximation (QHA), and phonons were calculated by sampling reciprocal lattice points by dividing one side of the Brillouin zone in half. Based on these models, the calculated relative Gibbs energies of formation for the undoped orthorhombic Sn_3_O_4_ are shown in Figure 1c. The zero value on the vertical axis corresponds to the formation energy of undoped Sn_3_O_4_. The results show that aluminum (Al)- and yttrium (Y)-cation-doped Sn_3_O_4_ at Sn^4+^ sites are more stable than nondoped Sn_3_O_4_. From the viewpoint of charge compensation in the Sn_3_O_4_ crystal, the introduction of trivalent cations, such as Al^3+^ or Y^3+^ ions, into the Sn^4+^ sites generated holes, as discussed later. Boron (B) and strontium (Sr) are stabilized at the Sn^2+^ site, whereas the introduction of transition metals such as iron (Fe), cobalt (Co), nickel (Ni), and copper (Cu) causes instability in the orthorhombic Sn_3_O_4_ crystal. On the other hand, niobium (Nb) and tantalum (Ta), which can exist as pentavalent ions, are relatively stable dopants compared with transition metals at Sn^4+^ sites. To verify whether the MLIP calculations were appropriate, we attempted to synthesize doped Sn_3_O_4_ with several cations (B, Y, Sr, Al, Nb, Ta, Fe, Co, Cu, and Ni) in powder form by a hydrothermal method, as described in the next section.

**Figure 1:**
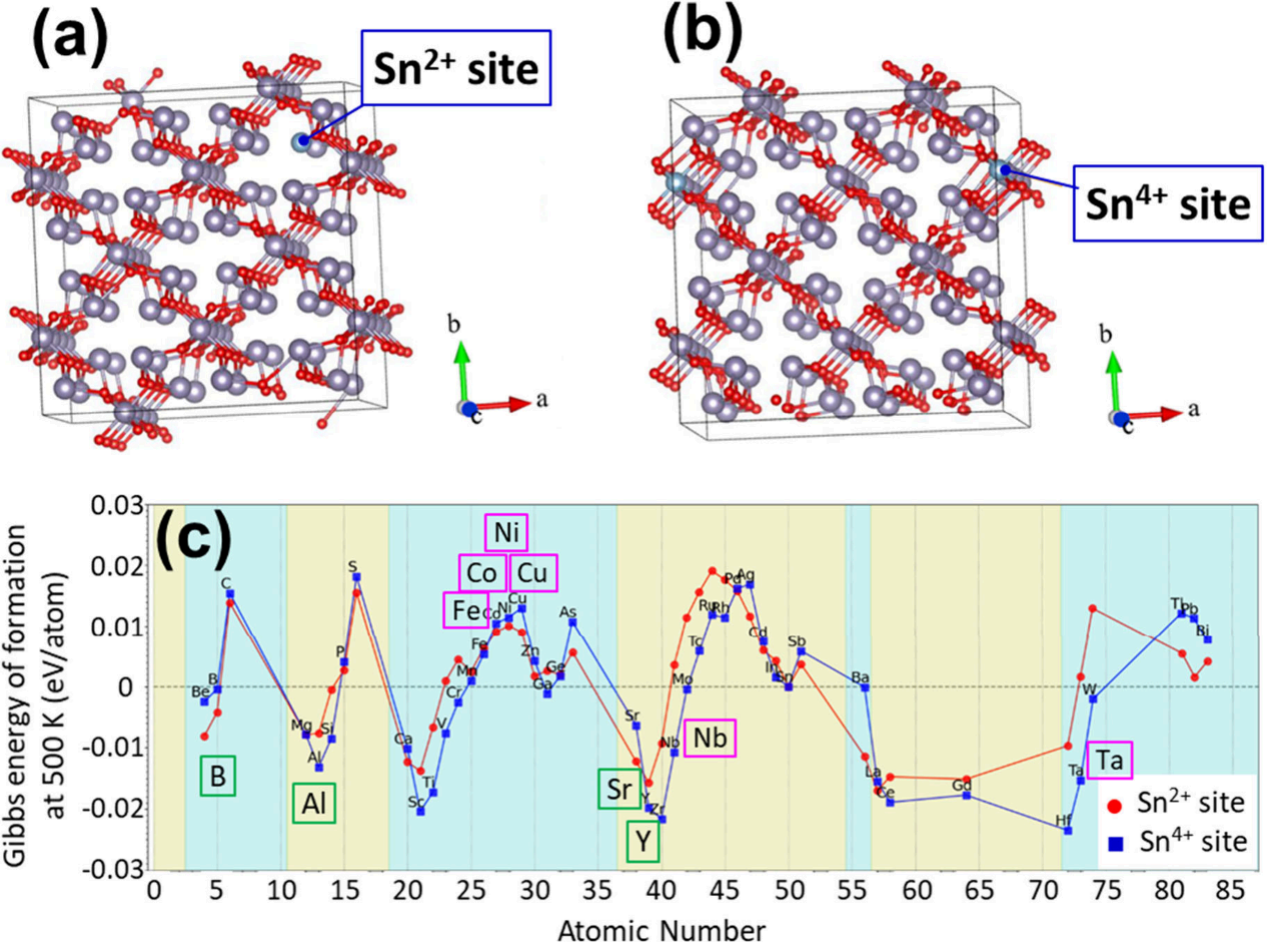
Cation screening by MLIP calculations. (a) model with Sn (gray) and O (red) atoms of doped Sn_3_O_4_ into Sn^2+^ site, (b) model of doped Sn_3_O_4_ into Sn^4+^ site, and (c) Gibbs energy of formation at 500 K for various doped orthorhombic Sn_3_O_4_ samples.

### Experimental Synthesis of Metal-Ion-Doped Orthorhombic Sn_3_O_4_

Powder forms of doped Sn_3_O_4_ were synthesized based on a recently reported hydrothermal method,^27^ in which foreign cation salts were introduced into a reactor, as listed in Table S1 in Supporting Information. In the synthesis, the foreign cation amount was set at 5 mol % versus the tin amount, where there is no guarantee that all the precursor cations could be doped into the Sn_3_O_4_ crystal, as described later. Figure 2 shows X-ray diffraction (XRD) patterns of various cation-doped Sn_3_O_4_ powder samples. Panel a shows the results for doped Sn_3_O_4_, which are stable groups suggested by MLIP calculations at both the Sn^2+^ and Sn^4+^ sites. The peaks observed in the undoped sample (closed circles) are all assigned to orthorhombic Sn_3_O_4_. Consistent with the results for the undoped sample, all peaks in the B-, Y-, Sr-, and Al-ion-doped Sn_3_O_4_ powder samples were assigned to orthorhombic Sn_3_O_4_ without the formation of any other crystal phase. Panel b shows the results for the samples that were the less stable groups predicted by the MLIP calculations. While the strongest peak of orthorhombic Sn_3_O_4_ was observed at 27.2°, with the (211) or (121) face^27^ the highest peak of most of the cation-doped samples shown in panel b was 26.8°, which was assigned to the (111) face of monoclinic Sn_3_O_4_ (PDF No. 20-1293).^4^ Almost all cation-doped Sn_3_O_4_ samples shown in panel b consist of a monoclinic Sn_3_O_4_ phase. In the case of the Cu-doped sample, peaks other than those of Sn_3_O_4_ were observed, which originated from the copper compound. For Nb-doping, the main crystal phase was cubic Sn_2_Nb_2_O_7_ (PDF No. 23-0593). Although the MLIP calculation results indicated that the Nb- or Ta-doped orthorhombic Sn_3_O_4_ was more stable at the Sn^4+^ site than the nondoped Sn_3_O_4_, their crystal structures were not in the orthorhombic phase. The precursors of these doped samples (NbCl_5_ and TaCl_5_) were highly reactive and vigorously hydrolyzed in aqueous solution before the hydrothermal nucleation process; thus, a single orthorhombic phase was not formed. Furthermore, if either the divalent or tetravalent site is thermodynamically unstable, it may induce instability in the desired structure along the reaction pathway during synthesis, potentially preventing its formation. Scanning electron microscopy (SEM) images of the doped orthorhombic Sn_3_O_4_ samples are shown in the Supporting Information (Figure S4), and the images exhibited rod-aggregated structures. The experimental trends in crystal formation are consistent with the MLIP calculations, indicating that our consideration of MLIP is an appropriate and efficient method for screening suitable dopants in a crystal. Hereafter, we compared the physicochemical properties of the stably doped samples.

**Figure 2:**
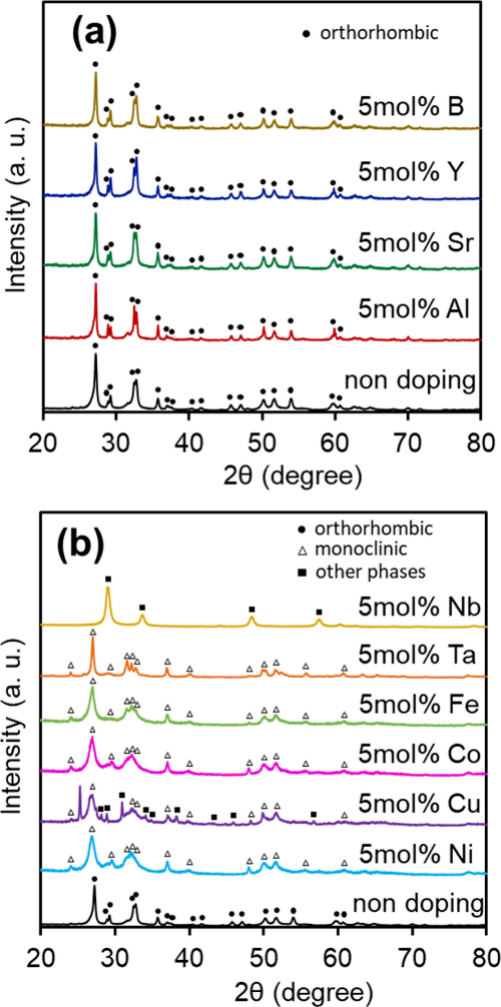
XRD patterns for various doped tin oxide samples with the doping density at 5 mol %: (a) B-, Y-, Sr-, and Al-doped samples; (b) Nb-, Ta-, Fe-, Co-, Cu-, and Ni-doped samples. Both panels include the XRD pattern of nondoped orthorhombic Sn_3_O_4_ powder. Closed circles, open triangles, and closed squares indicate orthorhombic Sn_3_O_4_, monoclinic Sn_3_O_4_, and other crystal phases of Sn_3_O_4_, respectively.

Figure 3a shows the ultraviolet–visible (UV–vis) absorption spectra recorded by the diffuse reflectance method for the stably doped orthorhombic Sn_3_O_4_ samples. The doped samples exhibited slight visible-light absorption tails over 500 nm. Photoemission yield spectroscopy (PYS) was conducted in air to estimate the valence band maximum (VBM) position using a previously described method.^27^ Figure 3b shows the PYS spectra of the stably doped orthorhombic Sn_3_O_4_ samples. The VBM position can be determined by the threshold photon energy versus the vacuum level, and the VBM of Al-doped Sn_3_O_4_ is close to that of undoped Sn_3_O_4_. It should be noted that the VBM value of the Sr-doped Sn_3_O_4_ was slightly shifted toward a large photon energy. Although the reason for the slight change in the absorption and PYS spectra upon doping is unclear, we compared their photocatalytic visible-light activities for hydrogen production.

**Figure 3:**
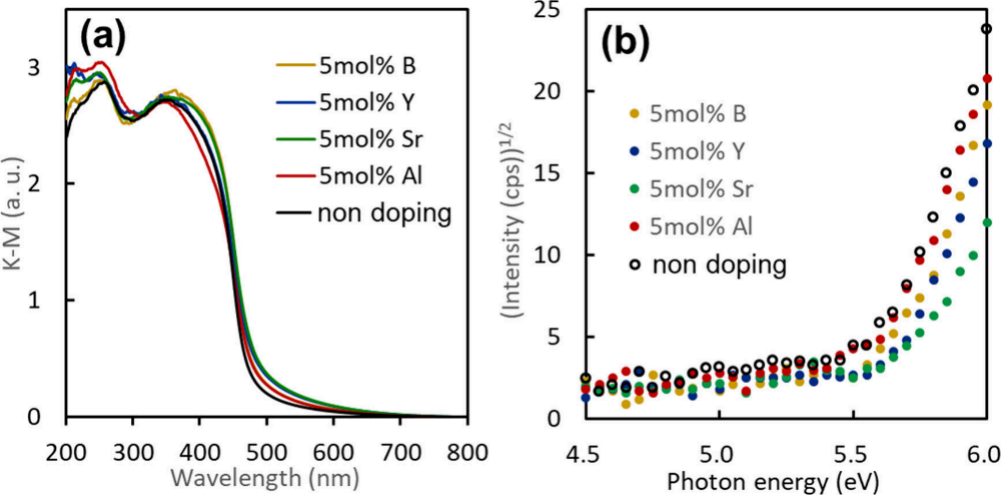
UV–vis absorption spectra (a) and PYS spectra (b) for doped orthorhombic Sn_3_O_4_.

The photocatalytic hydrogen production properties were determined using a closed quartz cell under visible-light irradiation (xenon lamp with short-wavelength cutoff filter λ = 422 nm) with a sacrificial ethanol agent, as described in the experimental details in the Supporting Information. In this experiment, we did not modify any cocatalyst promoters on the doped Sn_3_O_4_ samples. Figure 4 shows the amount of hydrogen gas produced in the headspace of the reactor. Notably, Al-doped orthorhombic Sn_3_O_4_ exhibited significant hydrogen production performance under visible light irradiation (λ > 422 nm). The photocatalytic activities of the other dopants were worse than those of undoped Sn_3_O_4_. The amount of gaseous hydrogen produced by Al-doped orthorhombic Sn_3_O_4_ was 16 times larger than that produced by undoped Sn_3_O_4_. Although the visible light absorption spectrum of Al-doped Sn_3_O_4_ was slightly shifted toward longer wavelengths, the large photocatalytic enhancement by Al doping cannot be explained by the absorbed photon number through a red shift. To investigate the improvement caused by Al doping, we fabricated thin films of Al-doped orthorhombic Sn_3_O_4_, because the film form is useful for photoelectrochemical studies.

**Figure 4:**
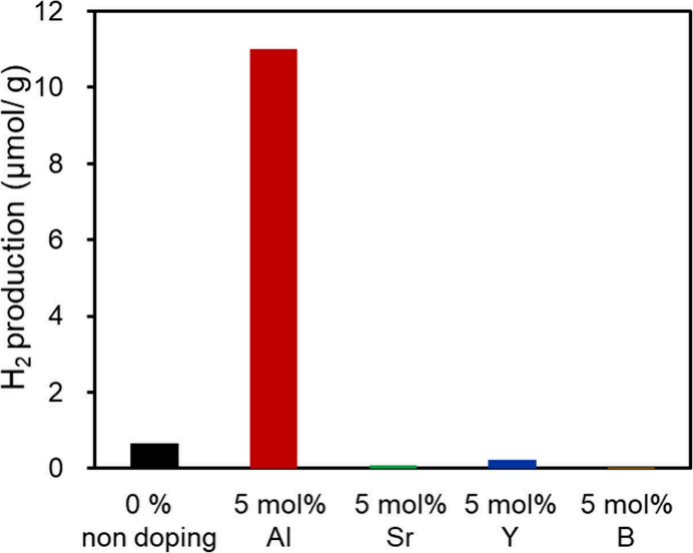
Photocatalytic hydrogen production activities of cation-doped orthorhombic Sn_3_O_4_ under visible-light irradiation (λ > 422 nm). Ethanol was added as a sacrificial agent, but no cocatalyst promoter was modified on the samples.

### Thin Films of Al-Doped Sn_3_O_4_ and Their Photoelectrochemical Properties

Films of orthorhombic Al-doped Sn_3_O_4_ were grown on fluorine-doped tin oxide (FTO)-coated substrates, based on our previous report.^13^ Detailed film fabrication methods are described in our Supporting Information. Similar to the powder fabrication process in the present study, we additionally introduced foreign cation salts (AlCl_3_·6H_2_O) in a reactor with various amounts (3, 5, 7, and 10 mol %). Figure 5 shows the XRD patterns of Al-doped Sn_3_O_4_ films with various doping amounts. The length of the *a*-axis (8.77166 Å) and that of the *b*-axis (8.75953 Å) in the orthorhombic Sn_3_O_4_ were very close,^27^ several peaks overlapped and could not be distinguished as a single diffraction peak in our X-ray diffractometer. In the case of the powder samples shown in Figure 2 (a), the strongest peak was seen at 27.2° with the (211) or (121) faces, whereas the peak intensity of the film samples for the (002) or (220) faces seen at 29.0° was much higher than those in the powder samples, as shown in Figure 5. These results indicate a strong crystal orientation in the film samples. Our previous study also suggested that the orthorhombic films were strongly oriented in the (001) direction.^13^ Heterogeneous nucleation and crystal growth of orthorhombic Sn_3_O_4_ are preferred on an FTO substrate rather than the free homogeneous nucleation in a solvent to form a powder.

**Figure 5:**
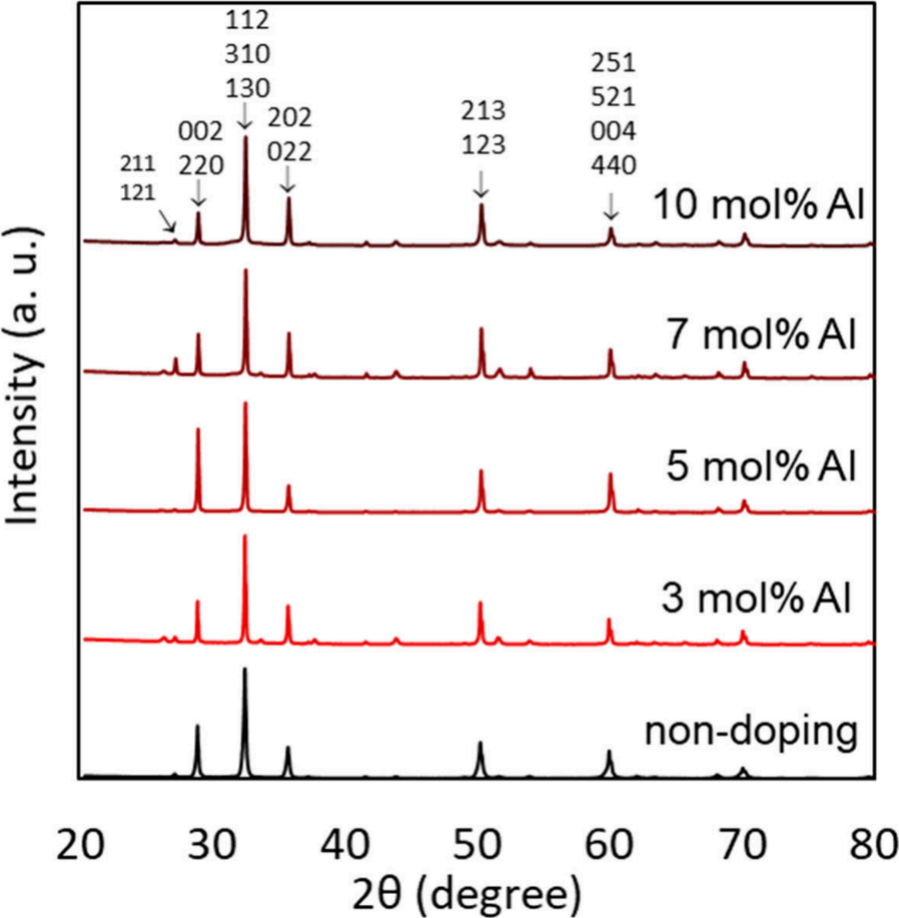
XRD patterns for Al-doped Sn_3_O_4_ films with various doping amounts. Miller indexes of major peaks are indicated. Major peak positions and their full width at half-maximum (FWMH) values of these peaks are listed in Table 1.

The XRD patterns of Al-doped Sn_3_O_4_ with various doping amounts are almost similar, as shown in Figure 5. Then, we carefully compared the shift of the peak positions and average full width at half-maximum (FWMH) values of the observed main peaks, and the results are shown in Table 1. When the doping amount of Al increased, the peak positions shifted toward larger 2θ values, indicating that the unit cell size was reduced by Al doping. As suggested by the MLIP calculations (Figure 1), the Al^3+^ ions were stably doped into the Sn^4+^ sites (blue squares) rather than the Sn^2+^ sites (red circles). The ionic size of Al^3+^ is 0.535 Å,^34^ which is smaller than that of Sn^4+^ (0.690 Å). Therefore, the unit cell size is reduced if Al^3+^ ions are substitutionally doped into the Sn^4+^ sites in orthorhombic Sn_3_O_4_. To clarify the Al doping site, we analyzed the intensity ratio of Sn^2+^ and Sn^4+^ atomic columns in high-angle annular dark-field scanning transmission electron microscopy (HAADF-STEM)^35^ images taken along the [100] orientation for both Al-doped and undoped samples (Figure S5 in Supporting Information). In the nondoped sample, the contrast intensities of Sn^2+^ and Sn^4+^ atomic columns were comparable. In contrast, in the 5% Al-doped sample, many atomic columns exhibited weaker contrast at the Sn^4+^ sites compared to the Sn^2+^ sites. These results provide strong evidence for the preferential substitution of Al into the Sn^4+^ sites in the orthorhombic Sn_3_O_4_ structure, highlighting the site-selective nature of Al doping and its role in enhancing crystallinity. In addition, the FWMH values of the Al-doped Sn_3_O_4_ samples were smaller than those of undoped Sn_3_O_4_ (0.240°), indicating that the crystallinity of orthorhombic Sn_3_O_4_ increased with Al doping. As discussed later, the introduction of Al^3+^ ions under hydrothermal reaction conditions accelerates the crystal growth of orthorhombic Sn_3_O_4_.

**Table 1:** 2 *θ* Values and Average Full Width at Half-Maximum (FWMH) Values for XRD Peaks of Al-Doped Sn_3_O_4_ Films

	2θ of 002 or 220 (deg)	2θ of 112, 310, or 130 (deg)	2θ of 202 or 022 (deg)	2θ of 213 or 123 (deg)	2θ of 251, 521, 004, or 440 (deg)	av FWMH (deg)
10 mol % Al	28.98	32.56	35.80	50.34	60.10	0.204
7 mol % Al	28.98	32.56	35.82	50.32	60.06	0.150
5 mol % Al	28.96	32.54	35.78	50.30	60.06	0.186
3 mol % Al	28.92	32.50	35.74	50.24	59.94	0.135
nondoped	28.92	32.50	35.74	50.24	59.96	0.240

Next, we investigated the chemical composition of the Al-doped Sn_3_O_4_ films using an energy-dispersive X-ray spectrometer (EDS) equipped with a scanning electron microscope. Figure 6 shows the relationship between the Al mol % measured by EDS and that introduced into the precursor solution in the hydrothermal reactor. Even though the nondoped Sn_3_O_4_ exhibited 0.15 mol % Al, it originated in the background level of the EDS signals. However, the amount of Al measured by EDS increased linearly as a function of the Al content in the precursor solution. When 5 mol % Al was introduced into the precursor solution, the analyzed amount was 0.40 mol %. These results indicate that the all Al^3+^ ions could not be introduced into the Sn_3_O_4_ films, but the Al^3+^ ions in the precursor solution were partially substituted to the Sn^4+^ sites. In other words, many Al^3+^ ions exist as free ions after hydrothermal reactions. As shown by the linear relationship in Figure 6, Al^3+^ ions were introduced into the orthorhombic Sn_3_O_4_ films by our hydrothermal process. We also measured the Al amount in the 5 mol % Al-doped Sn_3_O_4_ film by an inductively coupled plasma mass spectroscopy (ICP-MS), and the doping density of Al versus total metal elements (Al + Sn) was 0.16 mol %, which was close to those measured by EDS analysis.

**Figure 6:**
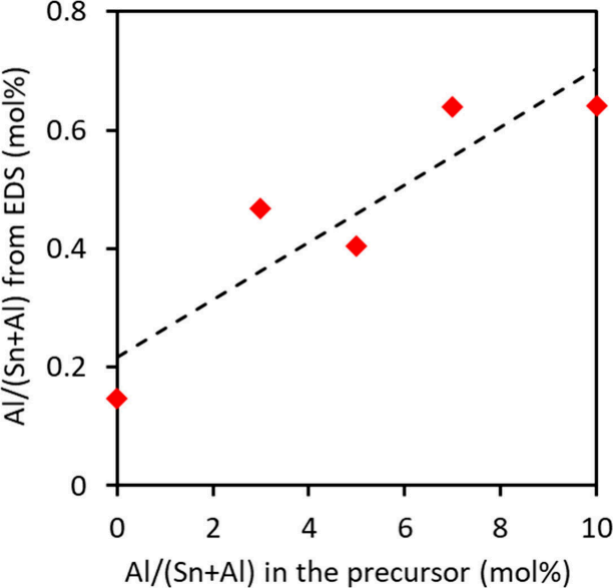
Relationship between experimental Al content measured by EDS and that in precursor solution. Al contents (mol %) were divided by the total (Al + Sn) amounts.

Figure 7 shows the SEM images of Al-doped orthorhombic Sn_3_O_4_ films with various Al doping amounts under the same magnification. In all cases, rectangular rod-like structures were observed in these samples. Notably, the rod size increases as the amount of Al doping increases. In the undoped sample, the size of the edge face was less than 500 nm. On the other hand, the size increased with Al doping, and was approximately 2 μm in the 10 mol % Al-doped Sn_3_O_4_, indicating that Al doping causes the high crystallinity of orthorhombic Sn_3_O_4_. These results are consistent with the FWMH results of the XRD patterns, in which the FWMH values decreased with Al doping.

**Figure 7:**
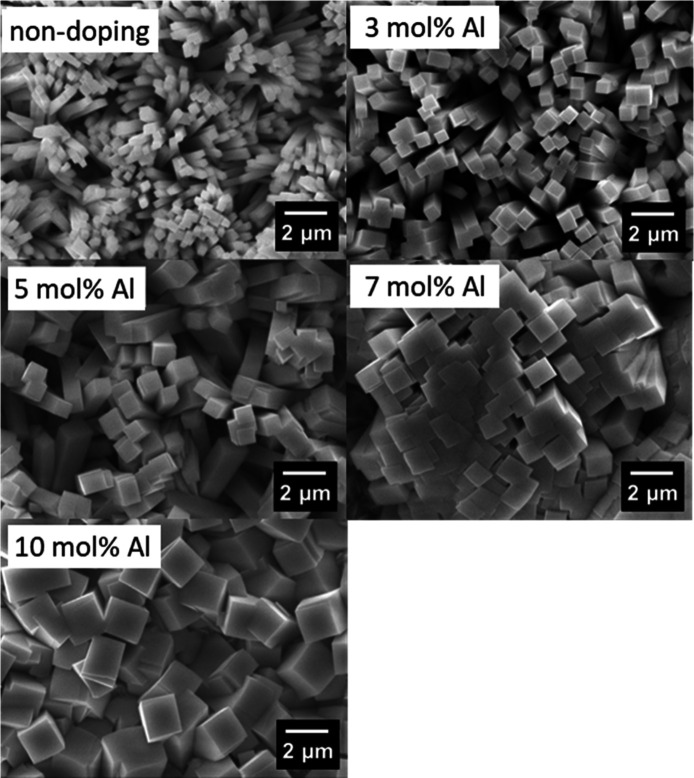
SEM images for Al-doped orthorhombic Sn_3_O_4_ films with various doping amounts.

To elucidate the origin of this particle size evolution, we note first that the rod-like morphology remained unchanged regardless of doping level, suggesting that the relative surface energies of the crystal facets were not significantly altered by Al incorporation. By contrast, the pronounced increase in particle size strongly indicates a reduction in the rate of nucleation. In this system, Sn^2+^ is stabilized as a Sn–citrate complex under ambient conditions, and under hydrothermal treatment at elevated temperature and alkaline pH, the complex gradually decomposes, followed by the formation of hydroxide intermediates, partial oxidation of Sn^2+^, eventually leading to the crystallization of Sn_3_O_4_.^4^ When Al^3+^ is introduced, its strong Lewis acidity leads to the consumption of OH^–^ ions in solution, which likely makes the hydrolysis of Sn^2+^ less favorable and thus delays nucleation. As a result, fewer nuclei are formed, and the remaining ones grow into larger rod-shaped crystals. During this process a portion of Al^3+^ was incorporated into the orthorhombic Sn_3_O_4_ lattice. Recent studies, including those by Takata et al.^36^ and Ogawa et al.,^37^ have highlighted the role of Al doping in defect engineering, and our results further suggest that Al can also act as an effective modifier to control the particle morphology of Sn_3_O_4_.

In addition to the influence of Al doping on the crystallinity of Sn_3_O_4_, charge carriers are generated because the valence number of foreign cations differs from that of tin ions. Thus, we evaluated the Mott–Schottky plots of the film electrodes, which are useful for discussing the carrier types of semiconductors.^38,39^ Figure S6 in the Supporting Information shows the Mott–Schottky plots for the Al-doped, Y-doped, B-doped, and nondoped Sn_3_O_4_ film electrodes. In the case of Al-doped orthorhombic Sn_3_O_4_, the slope is negative, whereas the other samples exhibit positive slopes. These results strongly suggest that Al doping causes p-type conductivity, whereas the others are n-type semiconductors. A previous study suggested that Al^3+^ ion doping into the Sn^4+^ sites in SnO_2_ generates holes to compensate for charge neutrality in the SnO_2_ crystal.^40,41^ We also conducted DFT calculations to determine the density of states of Al-doped orthorhombic Sn_3_O_4_ based on the model shown in Figure 1b, and the results are shown in the Supporting Information (Figure S7). With Al^3+^ ion doping, the Fermi level is located at the top of the valence band, which is formed from the oxygen and Sn^2+^ orbitals.^27^ These results strongly imply that Al doping promotes further oxidation at the Sn^2+^ sites, and Al-doped Sn_3_O_4_ becomes a p-type semiconductor with positive holes, consistent with the results of the Mott–Schottky plots.

Next, we evaluated the optical properties of the Al-doped Sn_3_O_4_ film electrodes. Figure 8a shows the Tauc plots derived from the optical UV–vis spectra. The estimated bandgap values of the undoped, 3, 5, 7, and 10 mol % Al-doped samples were 2.56, 2.54, 2.55, 2.52, and 2.56 eV, respectively. The bandgap values were not very different in these samples, even after Al doping; thus, the absorbed photon numbers under visible-light irradiation in the photocatalysis tests were not different in these samples. Figure 8b presents the PYS spectra of the samples. Notably, the VBM positions were shifted slightly toward smaller photon energies by Al doping. As discussed in the previous study,^27^ the top of the valence band of orthorhombic Sn_3_O_4_ is formed by hybridized orbitals of Sn^2+^ and oxygen, while Sn^4+^ forms deeper levels. As shown in Figure S7 in the Supporting Information, when one of the Sn^4+^ site is substituted with Al, the pDOS component of Al is slightly present around the top of the valence band, which would probably relate the shift of PYS data.

**Figure 8:**
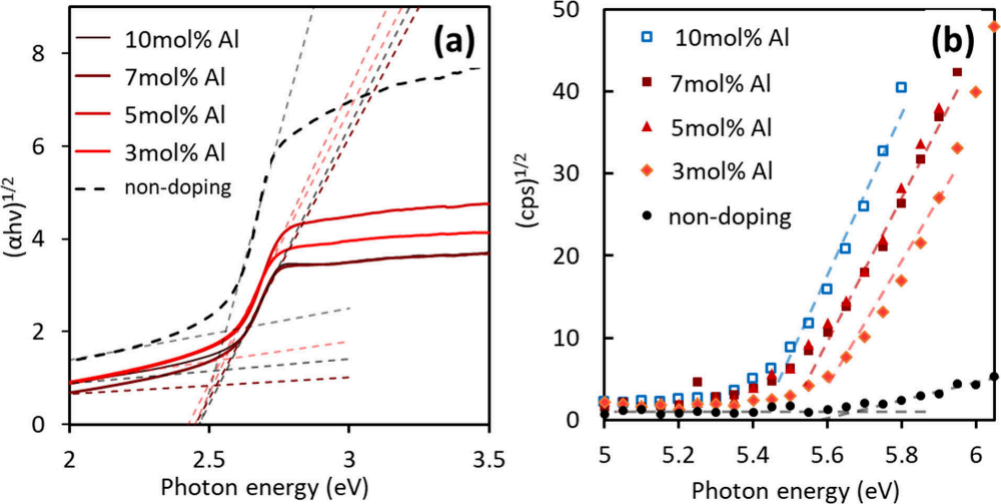
Tauc plots (a) and PYS (b) spectra for Al-doped orthorhombic Sn_3_O_4_ films with various doping amounts. Tauc plots are derived from optical UV–vis spectra recorded by a diffuse reflectance method. The estimated bandgap values for nondoping and 3, 5, 7, and 10 mol % Al-doped samples were 2.56, 2.54, 2.55, 2.52, and 2.56 eV, respectively.

We investigated the photocatalytic hydrogen production properties of the Al-doped Sn_3_O_4_ films under visible-light irradiation (λ > 430 nm). We confirmed that any hydrogen molecules was not generated from Sn_3_O_4_ under dark conditions (Figure S8 in the Supporting Information). Figure 9 shows the hydrogen generation activities under visible light irradiation, where ethanol was used as a sacrificial agent and no modifications were made to the cocatalyst promoter. Consistent with the powder results shown in Figure 4, the Al-doped films exhibited superior activity to the undoped Sn_3_O_4_ film. Among the samples, the 5 mol % Al-doped orthorhombic Sn_3_O_4_ film exhibited the highest activity. The stability of 5 mol % Al-doped Sn_3_O_4_ was confirmed by comparing XRD profiles before and after visible-light irradiation, and the pattern did not change after visible-light irradiation (Figure S9 in the Supporting Information). We also compared the photocatalytic performances of different Al contents of powder samples (Figure S10 in the Supporting Information), and the results revealed the similar trend with film results.

**Figure 9:**
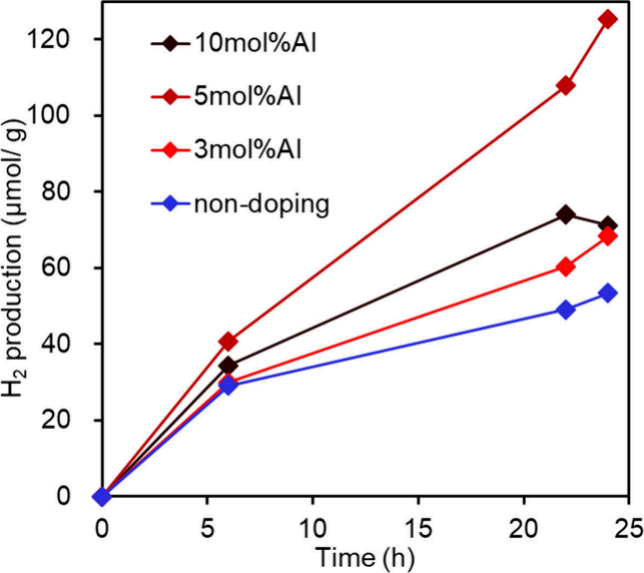
Photocatalytic hydrogen production activities of Al-doped orthorhombic Sn_3_O_4_ under visible-light irradiation (λ > 430 nm). Ethanol was added as a sacrificial agent, but no cocatalyst promoter was modified on the samples.

Here we discuss the reason for the improved photocatalytic activities by Al doping. We first note that the thermodynamic factor commonly discussed for photocatalysts, namely the redox driving force, plays only a minor role in the present case. This is because only minimal changes were observed between nondoped and 5 mol % Al-doped Sn_3_O_4_ in the positions of the conduction and valence bands, as evaluated by PYS and UV–vis spectroscopy (Figure 3). Therefore, we focus on changes in kinetic factors, such as charge supply, including charge generation, separation, and transport, and interfacial charge transfer where the redox reaction occurs. Here, we perform the photoelectrochemical impedance spectroscopy (PEIS) for nondoped and 5 mol % Al-doped Sn_3_O_4_. As shown in Figures S11–S14 in Supporting Information, multiple processes are observed in the impedance data. Among them, we focus on the low-frequency process that exhibits clear dependences on light intensity and applied potential, as this process is considered to be directly relevant to photo­(electro)­catalytic reactions (the remaining processes are discussed in the Supporting Information). This low-frequency process was fitted using an equivalent circuit composed of a resistance (*R*) and a constant phase element (CPE). It should be noted that the resistance *R* obtained from PEIS represents an apparent resistance derived from the differential response (d*V*/d*I*) under a small alternating current (AC) perturbation, rather than a pure kinetic charge-transfer resistance. Accordingly, variations in *R* reflect the combined effects of charge supply from the bulk and charge transfer at the surface, and its interpretation requires consideration of whether the system is limited by carrier supply or by surface charge-transfer processes. Figure 10 shows that the 5 mol % Al-doped sample exhibits a consistently smaller apparent charge-transfer resistance than the nondoped sample over the entire range of applied potentials and light intensities examined. This can be attributed to more effective utilization of photogenerated carriers that are successfully separated and reach the surface, indicating that Al doping improves the surface charge-transfer process.

**Figure 10:**
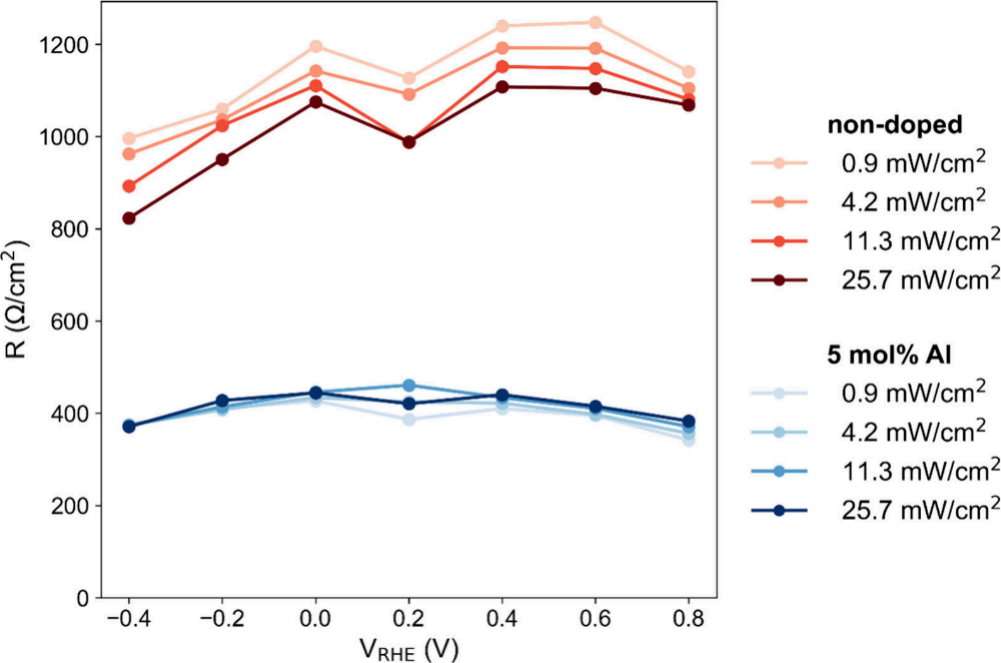
Resistance values obtained from PEIS analysis using the equivalent circuit model. The measurements were conducted under varying light intensities and applied potentials for both the nondoped and 5 mol % Al-doped samples. Detailed experimental conditions and analytical procedures are provided in the Supporting Information.

To further discuss the effect of Al doping, we next focus on the dependences on light intensity and applied potential in Figure 10. Compared to nondoped samples, it is observed that the doped sample shows little sensitivity to either light intensity or applied potential. This indicates that even when the number of photogenerated holes increases with increasing light intensity or applied potential, the charge-transfer process increases accordingly without introducing additional resistance. In other words, the Al-doped sample possesses a sufficiently high charge supply capability, so the reaction is not limited by carrier supply but instead governed by surface charge-transfer processes. In contrast, the nondoped sample exhibits pronounced dependences on both light intensity and applied potential. The decrease in resistance with increasing light intensity suggests that increasing the number of carriers available at the surface directly lowers the resistance, indicating that the reaction is limited by charge supply. This behavior implies that the intrinsic charge supply capability of the nondoped material is insufficient. On the other hand, an increase in resistance with increasing applied potential indicates that raising the potential does not lead to a corresponding increase in reaction current. Importantly, since the resistance still decreases upon increasing light intensity, this behavior cannot be attributed to a charge-transfer-limited regime. Instead, it suggests that the nondoped sample remains in a charge supply limited regime, where increasing the applied potential does not effectively enhance the number of reactive carriers at the surface. These results indicate that nondoped Sn_3_O_4_ suffers from an overall inefficient charge transfer and limited charge supply from the bulk, as compared to the Al-doped Sn_3_O_4_.

Finally, we discuss the consistency of the enhanced bulk charge supply with the morphology shown in Figure 7 and the facet-dependent roles reported in previous studies. According to the prior work, the top surface of the rod-like Sn_3_O_4_ particles corresponds to the (001) facet, which mainly serves as the reduction site, while the side facets, such as the (110) facet and its equivalents, are responsible for oxidation reactions.^13^ In the nondoped sample, the elongated morphology, consistent with the lower crystallinity summarized in Table 1, leads to longer transport distances for electrons and holes, which can reasonably contribute to inefficient carrier supply to the reactive surface sites. In contrast, the thicker rectangular morphology observed for the 5 mol % Al-doped sample, which is associated with better crystallinity, is expected to provide a more balanced transport pathway, facilitating improved charge supply. We also note that Al doping may influence exciton separation and charge mobility, which could further contribute to the observed enhancement. It should also be noted that the 10 mol % Al-doped sample showed inferior performance compared with the 5 mol % sample. This can be rationalized by the larger particle size at high doping levels, which increases the required diffusion length for photogenerated carriers beyond what the material can efficiently support. We also observed the SEM image of the yttrium (Y)-doped Sn_3_O_4_ film (Figure S15 in the Supporting Information), whose photocatalytic activity was worse than the Al-doped Sn_3_O_4_ film. The morphology of the Y-doped Sn_3_O_4_ film was different from that of the Al-doped Sn_3_O_4_ film. These results also suggest that crystallinity and morphological change are important factors to improve the photocatalytic activities of Sn_3_O_4_ films by Al doping. For further improvement of photocatalytic activities, cocatalyst modification is promising and is under investigation as a future study.

## Conclusions

Stable cation-doped orthorhombic Sn_3_O_4_ crystals were predicted using MLIP calculations, and B-, Al-, Sr-, and Y-doped orthorhombic Sn_3_O_4_ powders were synthesized via a hydrothermal reaction. Among the cation-doped orthorhombic Sn_3_O_4_ samples, the Al-doped Sn_3_O_4_ powder exhibited the highest photocatalytic activity for hydrogen production under visible light irradiation. We also developed thin films of Al-doped Sn_3_O_4_ and optimized the Al doping amount to achieve high photocatalytic activity. Among the various Al doping amounts, 5% Al-doped Sn_3_O_4_ exhibited the highest activity. Based on the kinetic study, its high visible-light photocatalytic activity is attributed to its high crystallinity and optimal morphology, rather than the change in the band structure by Al doping. The optimum Al-doped Sn_3_O_4_ facilitates better separation of the photogenerated carriers. We efficiently developed cation-doped orthorhombic Sn_3_O_4_ using MLIP calculations, and the developed material is expected to be applied as a visible light-sensitive photocatalyst. This study also showed that MLIP calculations are effective for developing unexplored materials with various functions.

## Experimental Section

See our Supporting Information in detail.

## Supplementary Material



## Notes

Experimental details, schematic illustration for the fabrication method of the orthorhombic Sn_3_O_4_ film, photographs of glass reactors for photocatalysis tests, spectra for visible light sources, SEM images, HAADF-STEM images, Mott–Schottky plots, calculated DOS, hydrogen generation under dark (control experiment), XRD patterns for before and after light irradiation, Al doping density dependence on photocatalytic activities, impedance analysis, and SEM images for Y- and Al-doped films (PDF)

## References

[1] Al-Hamdi A. M., Rinner U., Sillanpää M. (2017). Tin dioxide as a photocatalyst
for water treatment: A review. Process Safety
and Environmental Protection.

[2] Sun C., Yang J., Xu M., Cui Y., Ren W., Zhang J., Zhao H., Liang B. (2022). Recent intensification
strategies of SnO2-based photocatalysts: A review. Chemical Engineering Journal.

[3] Prakash K., Senthil Kumar P., Pandiaraj S., Saravanakumar K., Karuthapandian S. (2016). Controllable synthesis of SnO2 photocatalyst
with superior
photocatalytic activity for the degradation of methylene blue dye
solution. Journal of Experimental Nanoscience.

[4] Manikandan M., Tanabe T., Li P., Ueda S., Ramesh G. V., Kodiyath R., Wang J., Hara T., Dakshanamoorthy A., Ishihara S., Ariga K., Ye J., Umezawa N., Abe H. (2014). Photocatalytic Water Splitting under Visible Light by Mixed-Valence
Sn3O4. ACS Appl. Mater. Interfaces.

[5] Wang J., Umezawa N., Hosono H. (2016). Mixed Valence
Tin Oxides as Novel
van der Waals Materials: Theoretical Predictions and Potential Applications. Adv. Energy Mater..

[6] Yu X., Li C., Zhang J., Zhao L., Pang J., Ding L. (2024). Recent progress
on Sn3O4 nanomaterials for photocatalytic applications. International Journal of Minerals, Metallurgy and Materials.

[7] Berengue O. M., Simon R. A., Chiquito A. J., Dalmaschio C. J., Leite E. R., Guerreiro H. A., Guimarães F. E. G. (2010). Semiconducting
Sn3O4 nanobelts: Growth and electronic structure. J. Appl. Phys..

[8] Xia W., Qian H., Zeng X., Dong J., Wang J., Xu Q. (2017). Visible-Light Self-Powered Photodetector and Recoverable Photocatalyst
Fabricated from Vertically Aligned Sn3O4 Nanoflakes on Carbon Paper. J. Phys. Chem. C.

[9] Jose
Damaschio C., Berengue O. M., Stroppa D. G., Simon R. A., Ramirez A. J., Herwig Schreiner W., Chiquito A. J., Leite E. R. (2010). Sn3O4 single
crystal nanobelts grown by carbothermal reduction process. J. Cryst. Growth.

[10] Tanabe T., Hashimoto M., Mibu K., Tanikawa T., Gunji T., Kaneko S., Abe H., Miyauchi M., Matsumoto F. (2017). Synthesis
of Single Phase Sn3O4: Native Visible-Light-Sensitive Photocatalyst
with High Photocatalytic Performance for Hydrogen Evolution. J. Nanosci. Nanotechnol..

[11] Liu Y.-S., Yamaguchi A., Yang Y., Abe H., Ueda S., Tanabe T., Miyauchi M. (2021). Visible-Light-Induced CO2 Reduction
by Mixed-Valence Tin Oxide. ACS Applied Energy
Materials.

[12] Balgude S., Sethi Y., Kale B., Amalnerkar D., Adhyapak P. (2019). Sn3O4 microballs as highly efficient photocatalyst
for hydrogen generation and degradation of phenol under solar light
irradiation. Mater. Chem. Phys..

[13] Uchida S., Liu Y.-S., Yamaguchi A., Sekine Y., Yamaguchi K., Tanabe T., Miyauchi M. (2025). Synthesis
of Orthorhombic Sn3O4 Film
and Its Photocatalytic Visible-Light Activity. ACS Appl. Mater. Interfaces.

[14] Zeng D., Yu C., Fan Q., Zeng J., Wei L., Li Z., Yang K., Ji H. (2020). Theoretical and experimental research
of novel fluorine doped hierarchical Sn3O4 microspheres with excellent
photocatalytic performance for removal of Cr­(VI) and organic pollutants. Chemical Engineering Journal.

[15] Zhang L., Liu X., Zhang X., Zhang W., Ma J., Wang Q., Su S. (2023). Sulfur-doped Sn3O4 nanosheets for
improved photocatalytic performance. J. Alloys
Compd..

[16] Yang R., Ji Y., Wang L., Song G., Wang A., Ding L., Ren N., Lv Y., Zhang J., Yu X. (2020). Crystalline Ni-Doped
Sn3O4 Nanosheets for Photocatalytic H2 Production. ACS Applied Nano Materials.

[17] Liu H., Tan P., Yang L., Zhang M., Chen J., Ren R., Zhai H., Qi T., Pan J. (2023). In-doped Sn3O4 flower-like
nanosheets for efficient visible-light photocatalytic hydrogen production. Molecular Catalysis.

[18] Ma X., Shen J., Hu D., Sun L., Chen Y., Liu M., Li C., Ruan S. (2017). Preparation of three-dimensional
Ce-doped Sn3O4 hierarchical microsphere and its application on formaldehyde
gas sensor. J. Alloys Compd..

[19] Viswanath
N V., Yesuraj J., Kim K., Biswas K. (2024). Enhanced supercapacitive
performance in Cu doped Sn3O4 synthesized by a simple extract route. J. Alloys Compd..

[20] Li M., Li W., Lyu J., Ahmat Moussa M., Liu X., Fu Y., Ma H. (2021). Constructed
Co3O4-Sn3O4 hierarchical nanoflower-tree heterostructure
with boosting photoelectrocatalytic efficiency for water decontamination. Chemical Engineering Journal.

[21] Yuan N., Zhang X., Li B., Chen T., Yang X. (2023). Energy-Efficient
MIL-53­(Fe)/Sn3O4 Nanosheet Photocatalysts for Visible-Light Degradation
of Toxic Organics in Wastewater. ACS Applied
Nano Materials.

[22] Li C., Yu S., Dong H., Liu C., Wu H., Che H., Chen G. (2018). Z-scheme mesoporous photocatalyst constructed by modification of
Sn3O4 nanoclusters on g-C3N4 nanosheets with improved photocatalytic
performance and mechanism insight. Applied Catalysis
B: Environmental.

[23] Yang R., Song G., Wang L., Yang Z., Zhang J., Zhang X., Wang S., Ding L., Ren N., Wang A., Yu X. (2021). Full Solar-Spectrum-Driven Antibacterial
Therapy over Hierarchical Sn3O4/PDINH with Enhanced Photocatalytic
Activity. Small.

[24] Dai Z., Lian J., Sun Y., Li L., Zhang H., Hu N., Ding D. (2022). Fabrication of g-C3N4/Sn3O4/Ni
electrode for highly
efficient photoelectrocatalytic reduction of U­(VI). Chemical Engineering Journal.

[25] Yu X., Zhao Z., Ren N., Liu J., Sun D., Ding L., Liu H. (2018). Top or Bottom, Assembling Modules
Determine the Photocatalytic Property of the Sheetlike Nanostructured
Hybrid Photocatalyst Composed with Sn3O4 and rGO (GQD). ACS Sustainable Chem. Eng..

[26] Li S., Liu Z., Qu Z., Piao C., Liu J., Xu D., Li X., Wang J., Song Y. (2020). An all-solid-state Z-scheme NaNbO3-Au-Sn3O4
photocatalyst for effective degradation of carbofuran under sunlight
irradiation. J. Photochem. Photobiol., A.

[27] Liu Y.-S., Yamaguchi A., Yang Y., Aisnada A. N. E., Uchida S., Abe H., Ueda S., Yamaguchi K., Tanabe T., Miyauchi M. (2023). Synthesis
and Characterization of the Orthorhombic Sn3O4 Polymorph. Angew. Chem., Int. Ed..

[28] Rajeshwar K., Thomas A., Janáky C. (2015). Photocatalytic
Activity of Inorganic
Semiconductor Surfaces: Myths, Hype, and Reality. J. Phys. Chem. Lett..

[29] Fang W., Jiang Z., Yu L., Liu H., Shangguan W., Terashima C., Fujishima A. (2017). Novel dodecahedron BiVO4:YVO4 solid
solution with enhanced charge separation on adjacent exposed facets
for highly efficient overall water splitting. J. Catal..

[30] Takata T., Domen K. (2009). Defect Engineering of Photocatalysts by Doping of Aliovalent Metal
Cations for Efficient Water Splitting. J. Phys.
Chem. C.

[31] Asahi R., Morikawa T., Ohwaki T., Aoki K., Taga Y. (2001). Visible-Light
Photocatalysis in Nitrogen-Doped Titanium Oxides. Science.

[32] Takamoto S., Shinagawa C., Motoki D., Nakago K., Li W., Kurata I., Watanabe T., Yayama Y., Iriguchi H., Asano Y., Onodera T., Ishii T., Kudo T., Ono H., Sawada R., Ishitani R., Ong M., Yamaguchi T., Kataoka T., Hayashi A., Charoenphakdee N., Ibuka T. (2022). Towards universal neural network potential for material discovery
applicable to arbitrary combination of 45 elements. Nat. Commun..

[33] Jacobs R., Morgan D., Attarian S., Meng J., Shen C., Wu Z., Xie C. Y., Yang J. H., Artrith N., Blaiszik B., Ceder G., Choudhary K., Csanyi G., Cubuk E. D., Deng B., Drautz R., Fu X., Godwin J., Honavar V., Isayev O., Johansson A., Kozinsky B., Martiniani S., Ong S. P., Poltavsky I., Schmidt K. J., Takamoto S., Thompson A. P., Westermayr J., Wood B. M. (2025). A practical guide to machine learning interatomic potentials
- Status and future. Curr. Opin. Solid State
Mater. Sci..

[34] Liao S., Wang X., Yin H., Post J. E., Yan Y., Tan W., Huang Q., Liu F., Feng X. (2020). Effects of Al substitution
on local structure and morphology of lepidocrocite and its phosphate
adsorption kinetics. Geochim. Cosmochim. Acta.

[35] Abe E., Takakura H., Tsai A. (2001). Ho arrangement
in the Zn6Mg3Ho icosahedral
quasicrystal studied by atomic-resolution Z-contrast STEM. Journal of Electron Microscopy.

[36] Takata T., Jiang J., Sakata Y., Nakabayashi M., Shibata N., Nandal V., Seki K., Hisatomi T., Domen K. (2020). Photocatalytic water splitting with
a quantum efficiency of almost
unity. Nature.

[37] Ogawa K., Kavanagh S. R., Oba F., Walsh A. (2025). Defect Tolerance via
External Passivation in the Photocatalyst SrTiO3:Al. J. Am. Chem. Soc..

[38] Chiu Y.-H., Chang K.-D., Hsu Y.-J. (2018). Plasmon-mediated
charge dynamics
and photoactivity enhancement for Au-decorated ZnO nanocrystals. Journal of Materials Chemistry A.

[39] Chiu Y.-H., Lai T.-H., Chen C.-Y., Hsieh P.-Y., Ozasa K., Niinomi M., Okada K., Chang T.-F. M., Matsushita N., Sone M., Hsu Y.-J. (2018). Fully Depleted
Ti-Nb-Ta-Zr-O Nanotubes:
Interfacial Charge Dynamics and Solar Hydrogen Production. ACS Appl. Mater. Interfaces.

[40] Benouis C. E., Benhaliliba M., Mouffak Z., Avila-Garcia A., Tiburcio-Silver A., Ortega Lopez M., Romano Trujillo R., Ocak Y. S. (2014). The low resistive
and transparent Al-doped SnO2 films:
p-type conductivity, nanostructures and photoluminescence. J. Alloys Compd..

[41] Zhao J., Zhao X. J., Ni J. M., Tao H. Z. (2010). Structural,
electrical
and optical properties of p-type transparent conducting SnO2:Al film
derived from thermal diffusion of Al/SnO2/Al multilayer thin films. Acta Mater..

