# Supplementary material for: Computational and Experimental Realization of Metal-Ion-Doped Orthorhombic Sn3O4 for Visible-Light-Active Photocatalysis
Source: J Am Chem Soc. 2026 Feb 3;148(6):6036–44. doi: 10.1021/jacs.5c15962 (PMC12921868; doi:10.1021/jacs.5c15962)
Supplement: Supplementary file 1 [file ja5c15962_si_001.pdf]

Supporting Information

Computational and Experimental Realization of Metal-Ion-Doped Orthorhombic Sn_3O_4 for Visible-Light-Active Photocatalysis

Sho Uchida,[†] Yuta Sekine,[†] Yohei Cho,[†] Akira Yamaguchi,[†] Toyokazu Tanabe,[‡] Kenji Yamaguchi,[*,§] and Masahiro Miyauchi [*,†]

[†] Department of Materials Science and Engineering, School of Materials and Chemical Technology, Institute of Science Tokyo, Tokyo 152-8552, Japan
E-mail: mmiyauchi@ceram.titech.ac.jp

[‡] Department of Materials Science and Engineering, National Defense Academy, Kanagawa, 239-0811, Japan

[§] Innovation Center, Mitsubishi Materials Corporation, Ibaraki, 311-0102, Japan
E-mail: kyam@mmc.co.jp

Experimental Details

MLIP Calculations

Calculations were performed using PFP ver. 7.0.0 in a software-as-a-service (SaaS) environment called MATLANTIS. The accuracy of this version was validated by comparing its predictions of the energy above the hull for various inorganic crystals with DFT results, as reported on the MATLANTIS website (https://matlantis.com/en/product/validation/). As described in a previous report,[1] the training dataset for PFP was generated using DFT calculations performed with VASP, employing the PBE exchange-correlation functional. Seven volume points were sampled for QHA calculations. For each volume, full structural optimization, including the internal atomic positions, was performed, followed by phonon calculations. To compute the dynamical matrices required for phonon analysis of doped Sn_3O_4, a 2×2×2 supercell of the optimized structure was used. This supercell has a comparable volume to the 4×4×4 supercell of non-doped Sn_3O_4. Random atomic displacements (0.02 Å were applied to the supercell to evaluate the dynamical matrices. In addition, QHA calculations were conducted for stable elemental materials to evaluate their formation energies.

The DFT calculation of the density of states (DOS) for Al-doped Sn_3O_4, where Al substitutes for Sn^{4+} in the structure as depicted in Fig.1(b), optimized by PFP, was performed using VASP with the PBE exchange-correlation functional. The computational conditions were identical to those previously described.[2]

Synthesis of Cation-doped Sn_3O_4 Powder and Film

Orthorhombic Sn_3O_4 powder forms were synthesized by a hydrothermal method. 30 mmol of trisodium citrate dihydrate (Kanto Chemical Co., Inc.) was dissolved in 30 mL of water, then a total of 12 mmol of tin (II) chloride dihydrate (Kanto Chemical Co., Inc.) and impurity cation salt listed in **Table 2** were added to the solution and stirred until dissolution. The amount of cation salts was set at 5 mol % relative to the amount of tin. Then, 30 mL of 0.2 M NaOH was added with stirring for 30 min. After stirring, the solution was transferred into a 100 mL polytetrafluoroethylene (PTFE) reactor and sealed in a stainless-steel autoclave. Then, it was heated at 180 °C for 15 hours. After heating, the powder was collected by suction filtration with washing with distilled water and NaOH solution, followed by drying the powder at 40 °C.

Table S1. List of precursors for cation doping

cations	precursor salts	cations	precursor salts
strontium (Sr)	$SrCl_2 \cdot 6H_2O$	tantalum (Ta)	$TaCl_5$
aluminum (Al)	$AlCl_3 \cdot 6H_2O$	iron (Fe)	$FeCl_3 \cdot 6H_2O$
yttrium (Y)	$YCl_3 \cdot 6H_2O$	cobalt (Co)	$CoCl_2 \cdot 6H_2O$
boron (B)	$B(OH)_3$	nickel (Ni)	$NiCl_2 \cdot 6H_2O$
niobium (Nb)	$NbCl_5$	copper (Cu)	$CuCl_2 \cdot 2H_2O$

For the film preparation, we synthesized Al-doped Sn_3O_4, which exhibited the highest photocatalytic activity among the powders. Al-doped orthorhombic Sn_3O_4 films were grown on fluorine-doped tin oxide (FTO)-coated substrates in a manner similar to the powder fabrication. 20 mmol of trisodium citrate dihydrate was dissolved in 20 mL of water, then totally 8 mmol of tin (II) chloride dihydrate (Kanto Chemical Co., Inc.) and aluminum (III) chloride were added to the solution in various amounts (3, 5, 7, and 10 mol% tin). Then, 20 mL of a 0.2 M sodium hydroxide (NaOH) aqueous solution was added to the solution and stirred for 30 min. The solution became transparent on stirring. FTO substrates (3 cm × 4 cm in size) were washed with a mixture of water and ethanol with ultrasonication and fixed on a polytetrafluoroethylene (PTFE) bridge using polyimide tape in a 100 mL PTFE reactor (San-Ai Kagaku Co. Ltd., HUT-100), as shown in the Supporting Information (**Figure S1**). The precursor solution was transferred to a polytetrafluoroethylene (PTFE) reactor and sealed in a stainless-steel autoclave (San-Ai Kagaku Co., Ltd., HU-100). The autoclave was heated by an electric furnace at 180 °C for 15 hours. After the hydrothermal processing, the autoclave was quenched in water at room temperature. The film was washed with distilled water and an aqueous NaOH solution to remove impurities, and then it was dried at 40 °C.

Characterizations

The X-ray diffraction (XRD) patterns of Sn_3O_4 films were recorded using an out-of-plane method (MiniFlex, Rigaku Co.) with CuKα radiation. The range of the 2θ angle was from 20° to 80°. XRD patterns of powder samples were recorded using an out-of-plane method (Smart-lab, Rigaku Co.) with CuKα radiation. The morphologies of the powders and films were observed using scanning electron microscopy (SEM, JEOL JCM-7000). The amount of doped Al was measured using an energy-dispersive

spectroscopy (EDS) equipped with an SEM apparatus. Atomic microstructures were examined by means of scanning transmission electron microscopy (STEM). High-angle annular darkfield (HAADF) images were taken by a JEOL JEM-ARM200F. Chemical compositions of Al-doped Sn_3O_4 films were measured using an inductively coupled plasma mass spectroscopy (ICP-MS, Agilent Technologies, 7700x). UV-visible (UV-vis) diffuse reflectance spectra were recorded using a spectrometer (V-770, JASCO Co., Ltd.) equipped with an integration sphere unit. A mullite plate was used for the baseline calibration of the film samples, whereas barium sulfate was used for the powder samples. Valence-band estimation was performed using photoemission yield spectroscopy (PYS, Riken Keiki AC-3).

Electrochemical Mott-Schottky Plots of Film Electrodes

Electrochemical measurements were performed in a glass cell using the three-electrode method with a potentiostat (HZ-Pro, Hokuto-Denko). A Sn_3O_4 photoelectrode, a Pt plate, and an Ag/AgCl electrode were used as the working, counter, and reference electrodes, respectively. All electrochemical experiments were carried out in a 0.5 M sodium sulfate aqueous solution (pH= 7.66). Mott-Schottky plots were obtained using a frequency response analyser (FRA) equipped with a potentiostat (HZ-Pro, Hokuto-Denko). The potential change step was 50 mV, and the amplitude of the alternating current (AC) voltage was 20 mV in the Mott-Schottky plot. The area of the film exposed to the electrolyte solution was 1 cm^2 for each electrode.

Photocatalytic Activities of Powder and Films

The photocatalytic activities of the powder and film samples were evaluated in quartz glass reactors, as shown in the Supporting Information (**Figure S2**). For powder evaluation, 20 vol% of aqueous ethanol solution (3 mL) was added to a quartz cell, to which 20 mg of the powder photocatalyst was added. The solution was stirred during the photocatalytic tests. Before the photocatalytic tests, argon (Ar) gas was bubbled through the solution for 30 min. Visible light was irradiated through a side face using a 150 W xenon lamp (LA-410UV-03, Hayashi-Repic Co., Ltd.) equipped with the long-pass (short-cutoff) filter ($\lambda > 422$ nm, Asahi-Spectra Co. Ltd.) for 5 h. The visible light spectrum in this experiment is shown in the Supporting Information (**Figure S3 (a)**).

To evaluate the film systems, a Sn_3O_4 film was placed at the bottom of the reactor, and 40 mL of 20 vol% aqueous ethanol solution was added as a sacrificial agent. Before the photocatalytic tests, argon (Ar) gas was bubbled through the solution for 30 min. After the Ar bubbling, visible light was irradiated to the reactor using a 150

W xenon lamp (LA-410UV-03, Hayashi-Repic Co., Ltd.) equipped with the long-pass (short-cutoff) filter ($\lambda > 430$ nm, Asahi-Spectra Co. Ltd.). The visible light spectrum in this experiment is shown in the Supporting Information (**Figure S3 (b)**). Visible light was irradiated from the bottom of the reactor and passed through an FTO substrate. The H_2 concentration was measured using a gas chromatograph equipped with a dielectric barrier discharge ionization detector ("GC-BID", GC-2010, SHIMAZU Co. Ltd., Japan).

Photoelectrochemical Impedance Spectroscopy (PEIS) Measurement

PEIS measurements were performed using a three-electrode system. The non-doped and 5 mol% Al-doped samples were used as the working electrodes, a platinum wire as the counter electrode, and an Ag/AgCl electrode as the reference. The solution pH was controlled using a 0.1 M sodium phosphate (NaPi) buffer prepared in 0.2 M sodium sulfate (Na_2SO_4), and 20 vol% ethanol as a sacrificial reagent. The AC perturbation amplitude was set to 50 mV, and the frequency was swept from 10^6 Hz to 0.1 Hz.

For the equivalent circuit analysis, only the lowest-frequency process, which showed dependence on both light intensity and applied potential, was fitted using a parallel element consisting of a resistance and a constant-phase element (CPE).
The CPE impedance was expressed as:

$$Z_{CPE} = \frac{1}{Q(\mathrm{j}\omega)^n}$$

where Q is the CPE constant, n is the exponential factor, j is the imaginary unit, and omega is the angular frequency.

References in Supporting Information

1. Takamoto, S.; Shinagawa, C.; Motoki, D.; Nakago, K.; Li, W.; Kurata, I.; Watanabe, T.; Yayama, Y.; Iriguchi, H.; Asano, Y.; Onodera, T.; Ishii, T.; Kudo, T.; Ono, H.; Sawada, R.; Ishitani, R.; Ong, M.; Yamaguchi, T.; Kataoka, T.; Hayashi, A.; Charoenphakdee, N.; Ibuka, T., Towards universal neural network potential for material discovery applicable to arbitrary combination of 45 elements. *Nature Communications* **2022,** *13* (1), 2991.

2. Liu, Y.-S.; Yamaguchi, A.; Yang, Y.; Aisnada, A. N. E.; Uchida, S.; Abe, H.; Ueda, S.; Yamaguchi, K.; Tanabe, T.; Miyauchi, M., Synthesis and Characterization of the Orthorhombic Sn_3O_4 Polymorph. *Angewandte Chemie International Edition* **2023,** *62* (17), e202300640.

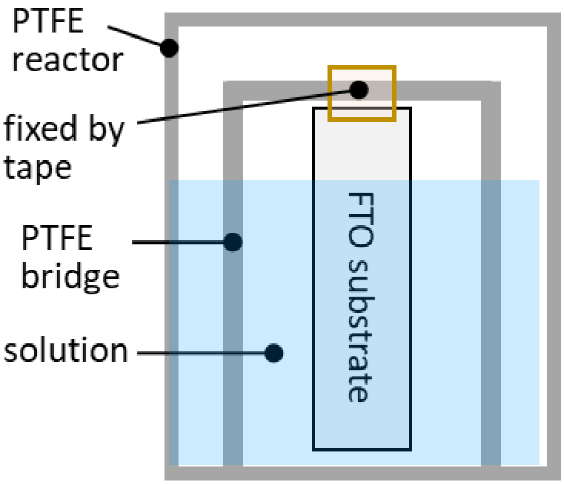

Figure S1. Schematic illustration for the fabrication method of the orthorhombic Sn_3O_4 film. A polytetrafluoroethylene (PTFE) reactor was used for the synthesis.

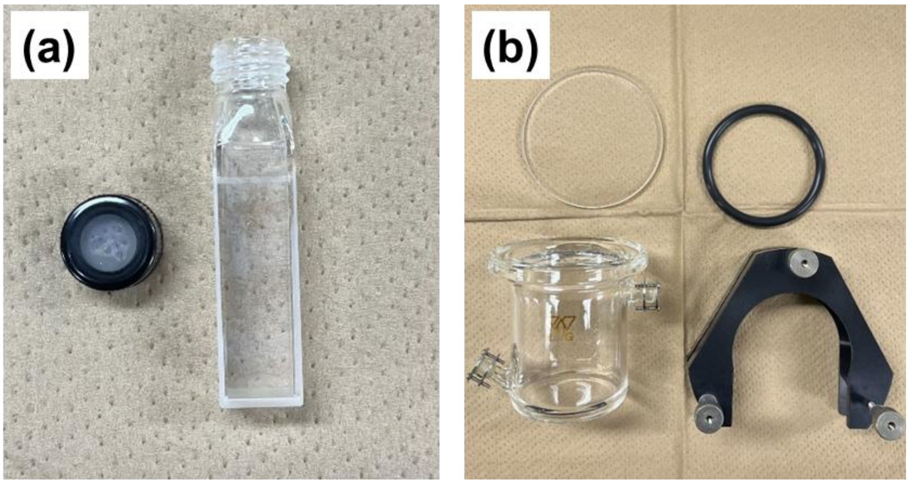

Figure S2. Photos of glass reactors for photocatalysis tests. (a) quarts cell for the evaluation of powder samples, (b) glass cell with a quartz window for the evaluation of film samples.

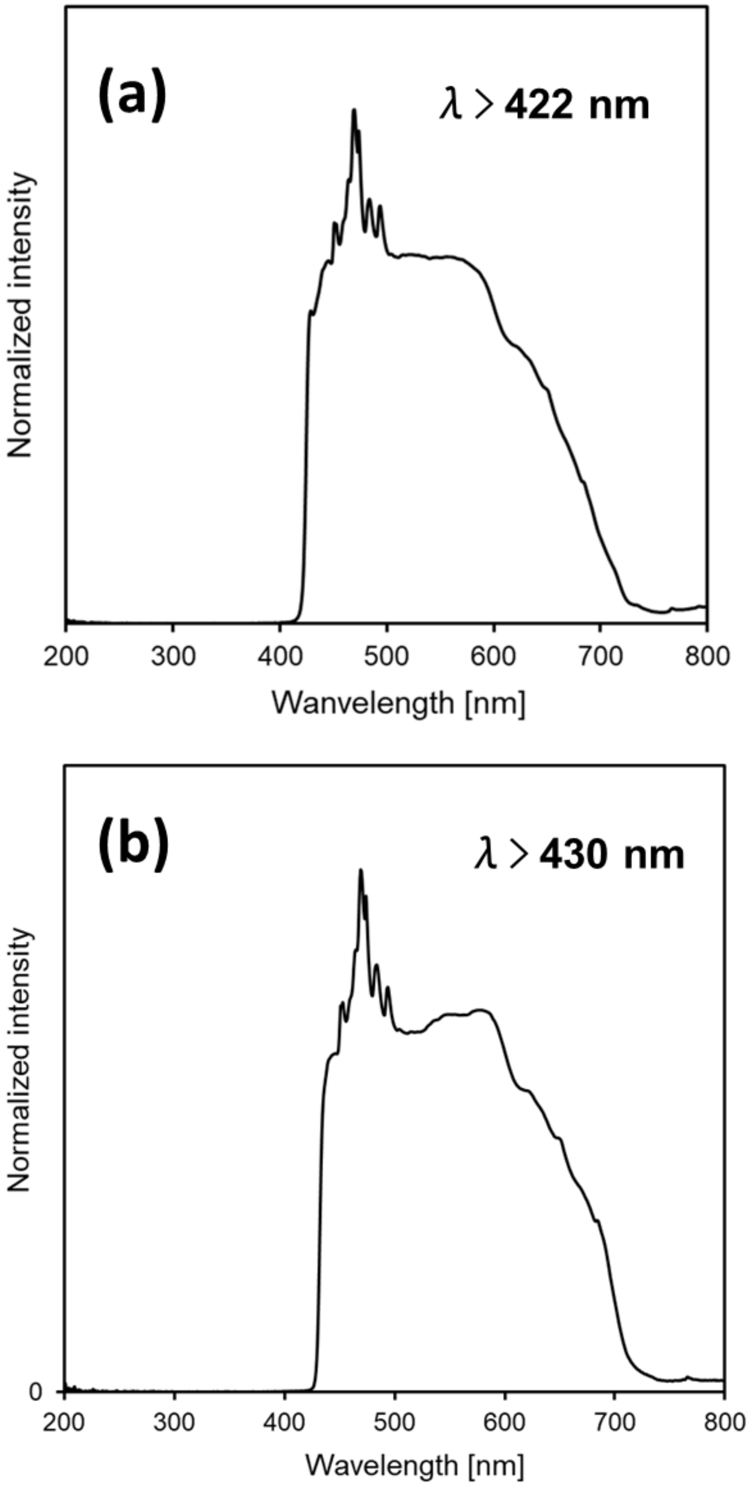

Figure S3. Spectra of the visible light source. A 150 W xenon lamp equipped with the long-pass (short-cutoff) filter, (a) $\lambda > 422$ nm and (b) $\lambda > 430$ nm.

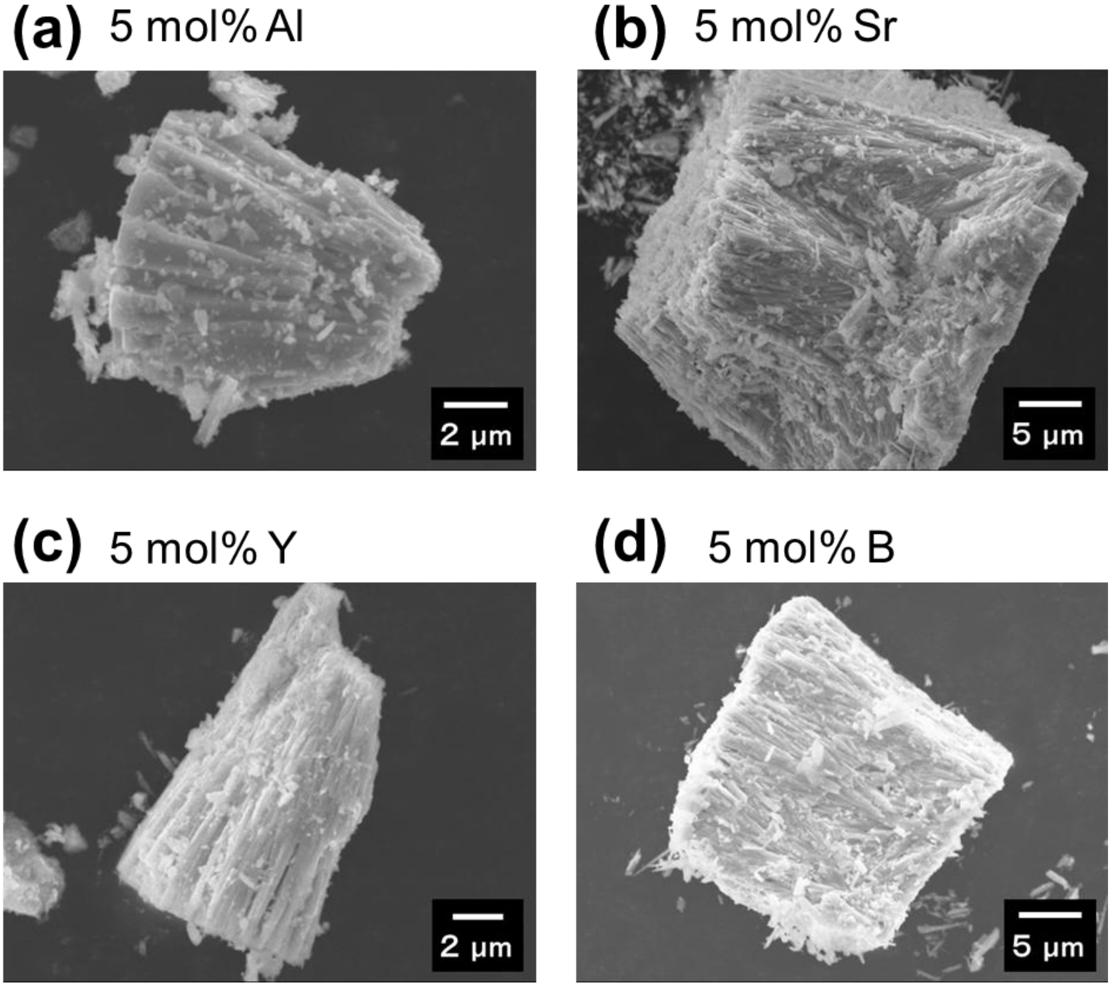

Figure S4. SEM images of doped orthorhombic Sn_3O_4 samples. (a) Al, (b) Sr, (c) Y, and (d) B ions with 5 mol%, respectively.

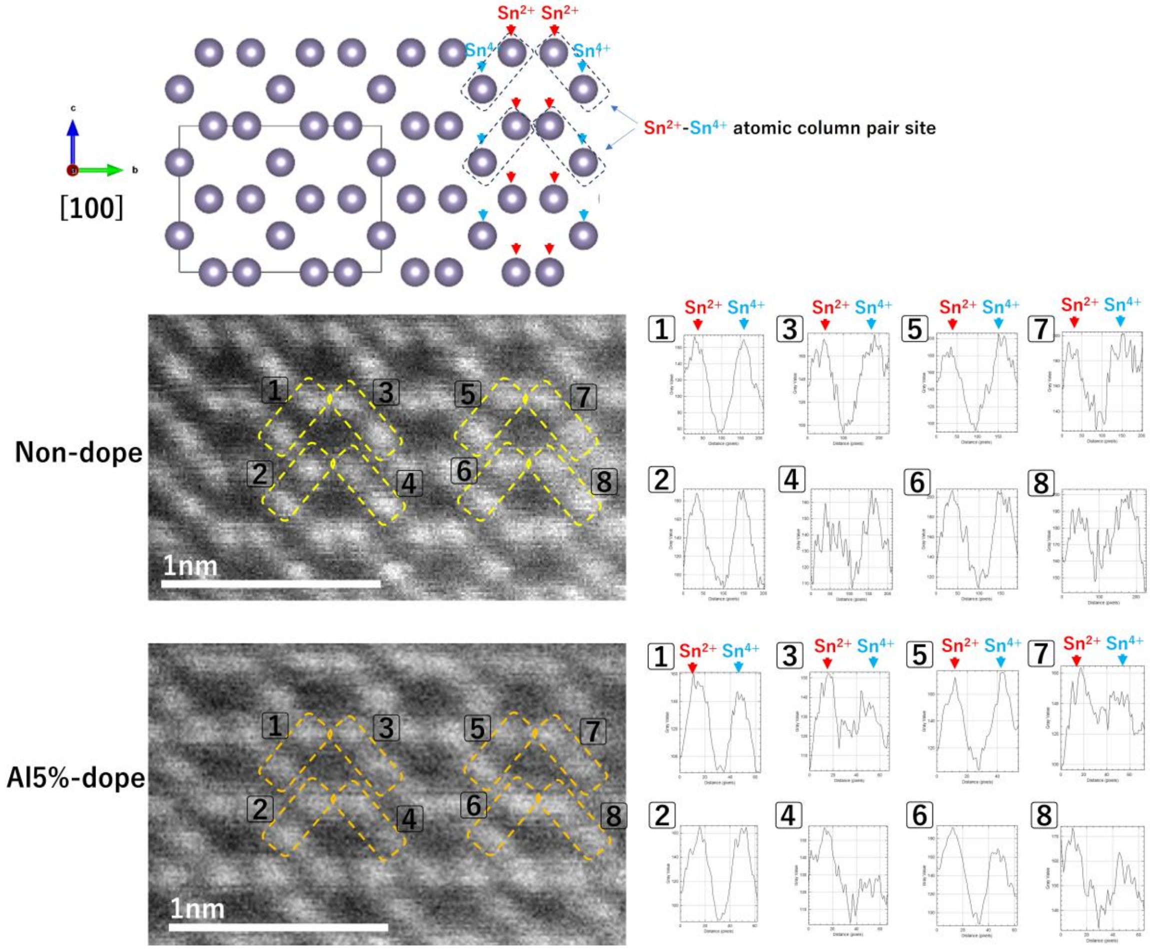

Figure S5. HAADF-STEM images of non-doped and 5% Al-doped Sn_3O_4 projected along the [100] direction. The corresponding image contrast illustrates the distinction between Sn^{2+} and Sn^{4+} atomic column pair sites.

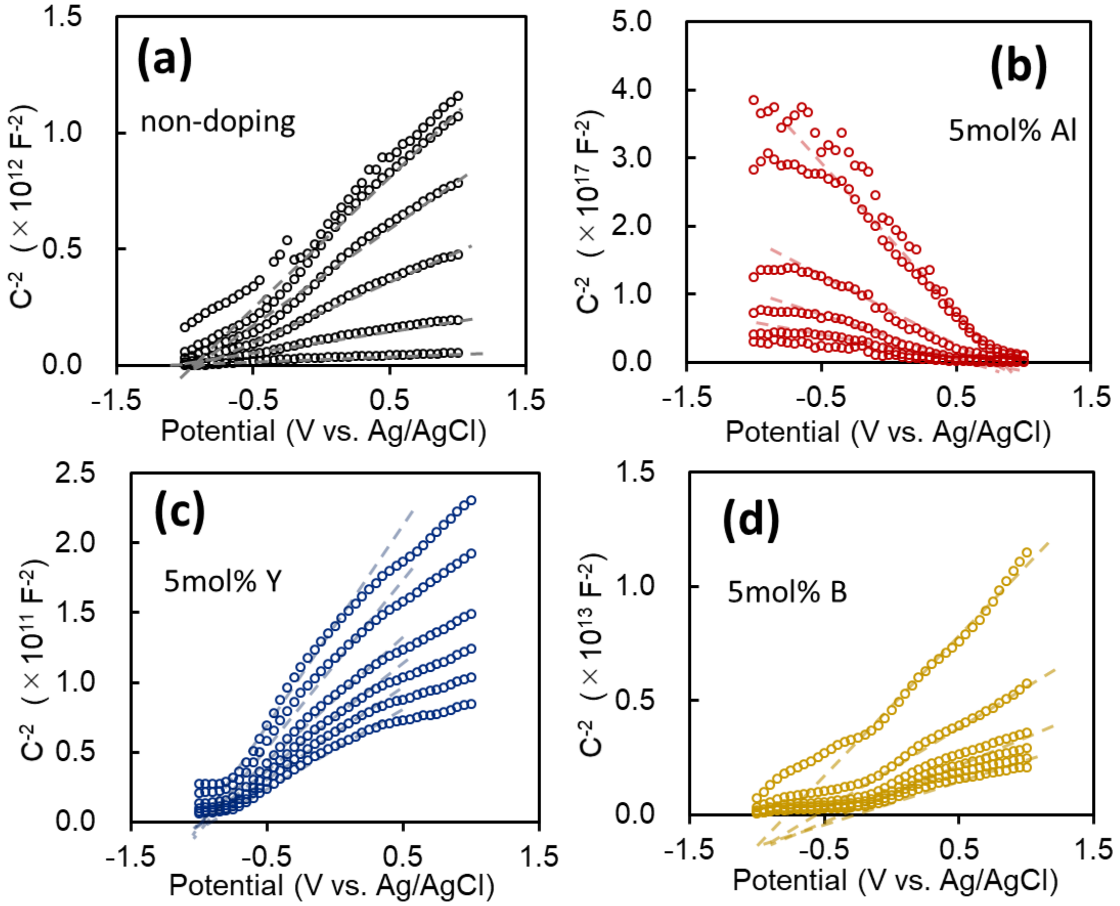

Figure S6. Mott-Schottky plots of non-doping (a), Al-doped (b), Y-doped (c), and B-doped Sn_3O_4 film electrodes. Doping amount was 5 mol% for all doped samples.

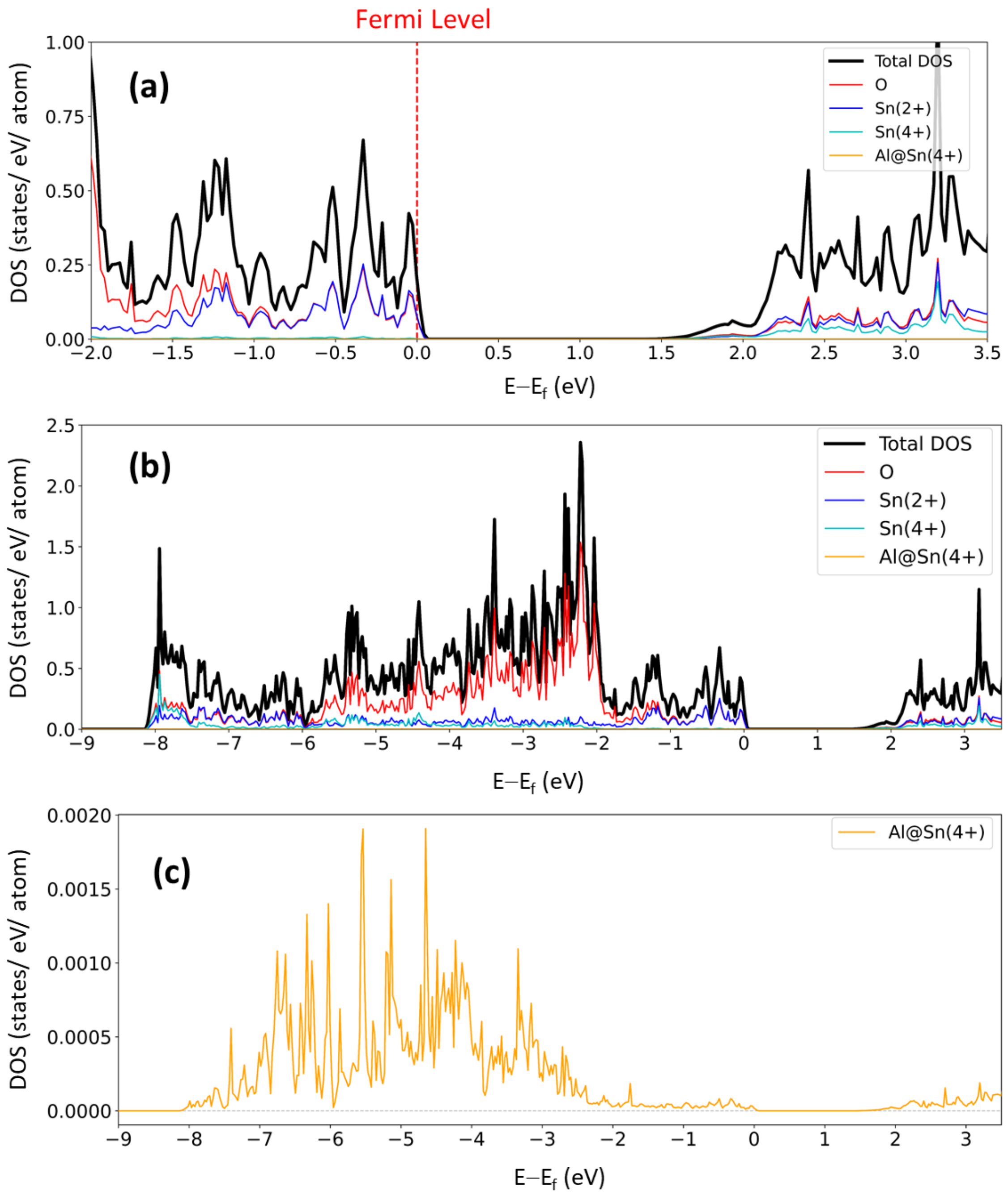

Figure S7. Density of states (DOS) of Al-doped orthorhombic Sn_3O_4. (a) from -2.0 to 3.5 eV around bandgap region, (b) from -9 to 3 eV, and (c) data for Al@Sn(4+), respectively.

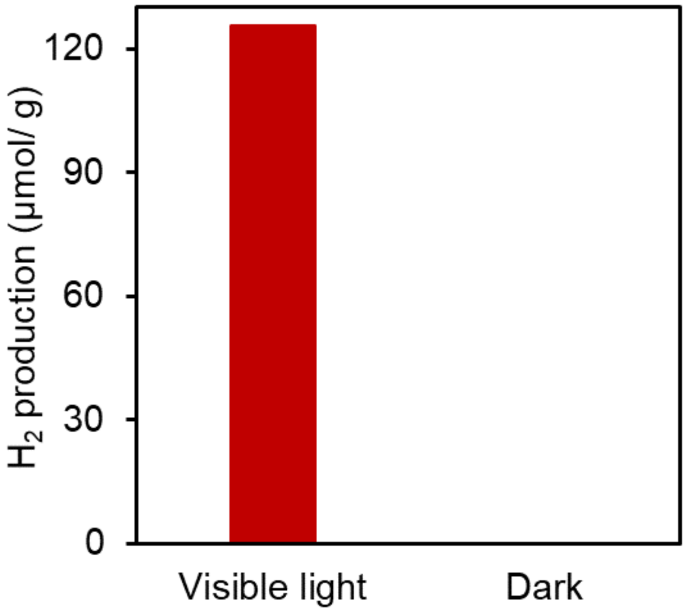

Figure S8. Generated hydrogen amount from 5 mol% Al-doped Sn_3O_4 under visible light irradiation and under the dark condition.

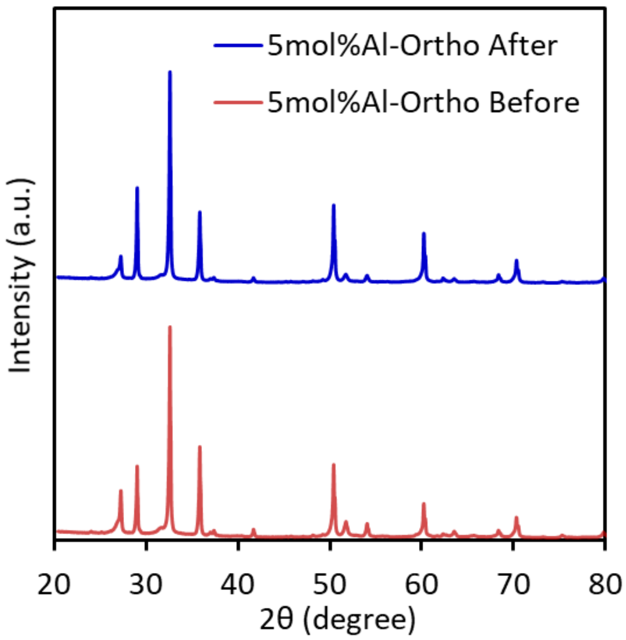

Figure S9. XRD patterns of 5 mol% Al-doped Sn_3O_4 before light irradiation (red line) and after light irradiation (blue line).

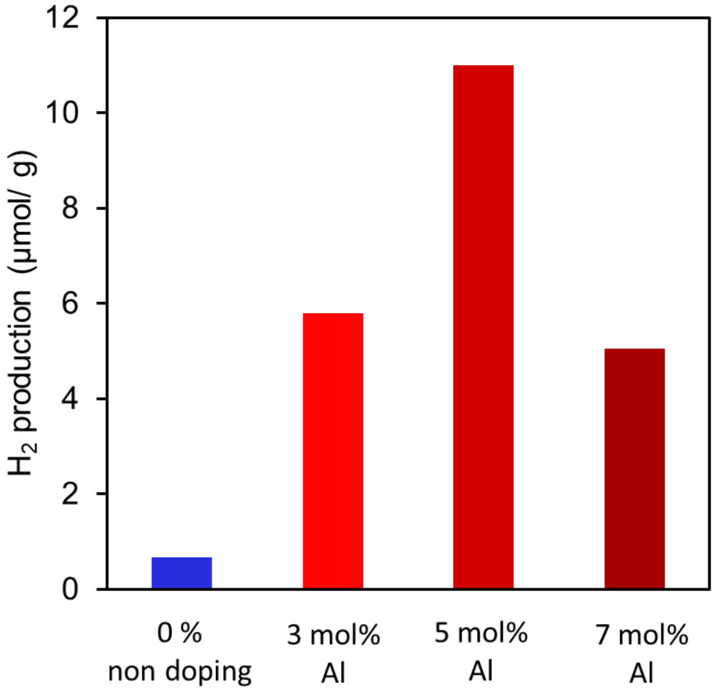

Figure S10. Photocatalytic hydrogen production activities of Al-doped Sn_3O_4 powder samples with different doping densities under visible light irradiation ($\lambda > 422$ nm) for 5 hours. Ethanol was added as a sacrificial agent, but no cocatalyst promoter was modified on the samples.

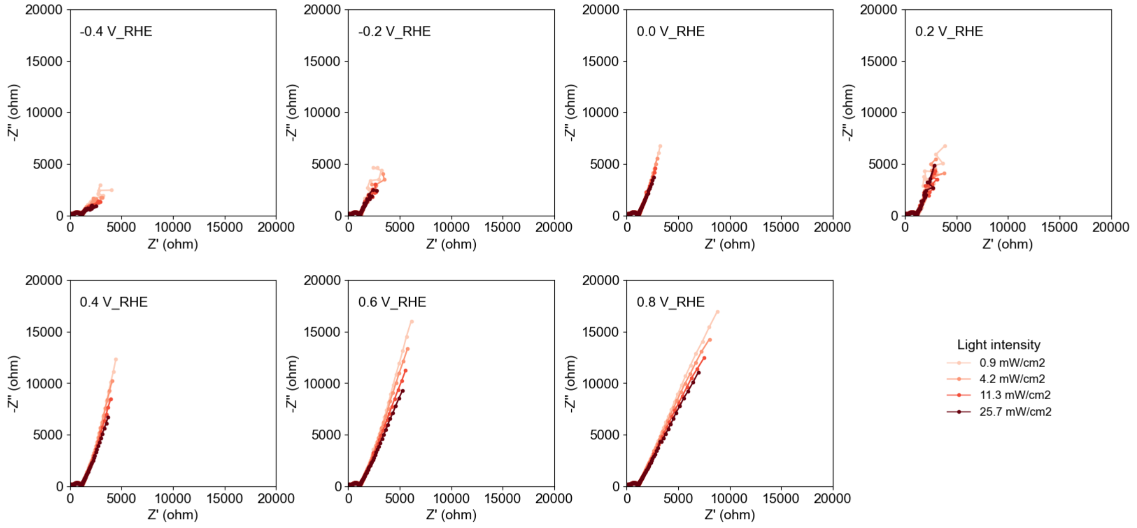

Figure S11. Nyquist plots of PEIS for non-doped Sn_3O_4 measured under different light intensities and applied potentials. The process on the right side of the plot (lower-frequency region), which shows dependence on both applied potential and light intensity, was fitted using a CPE (constant-phase element).

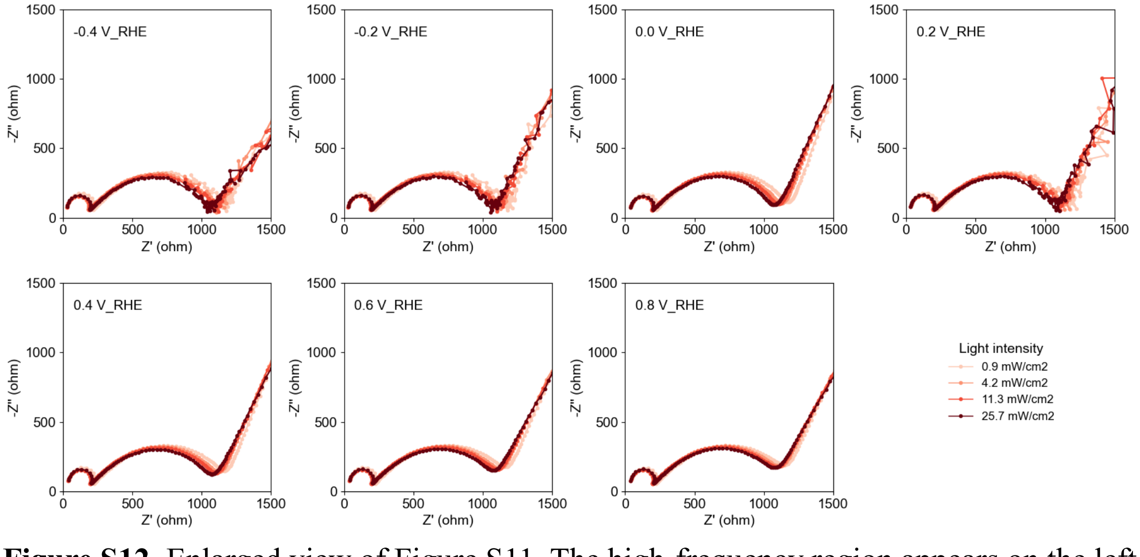

Figure S12. Enlarged view of Figure S11. The high-frequency region appears on the left side.

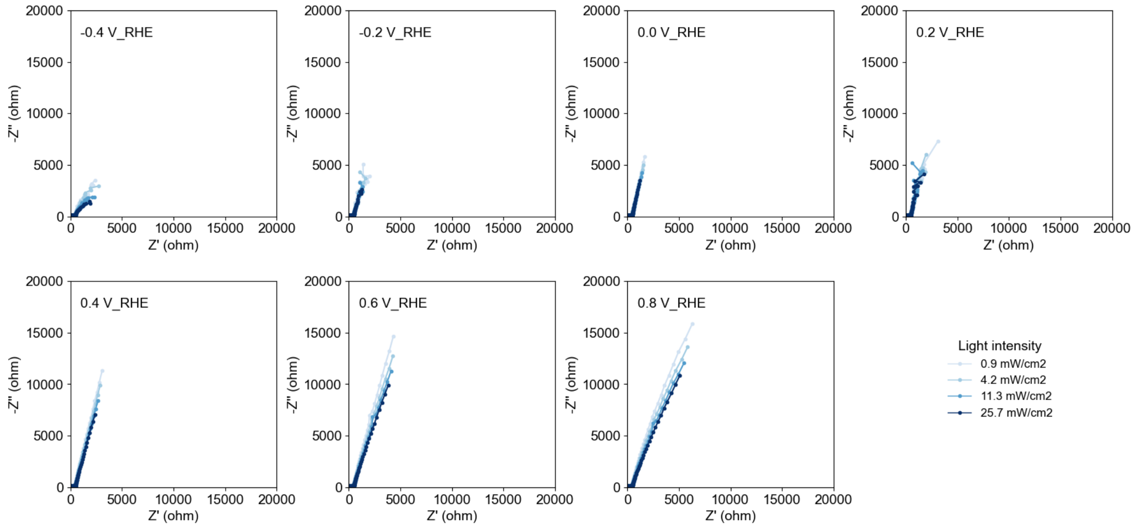

Figure S13. Nyquist plots of PEIS for 5 mol% Al-doped Sn_3O_4 measured under different light intensities and applied potentials.

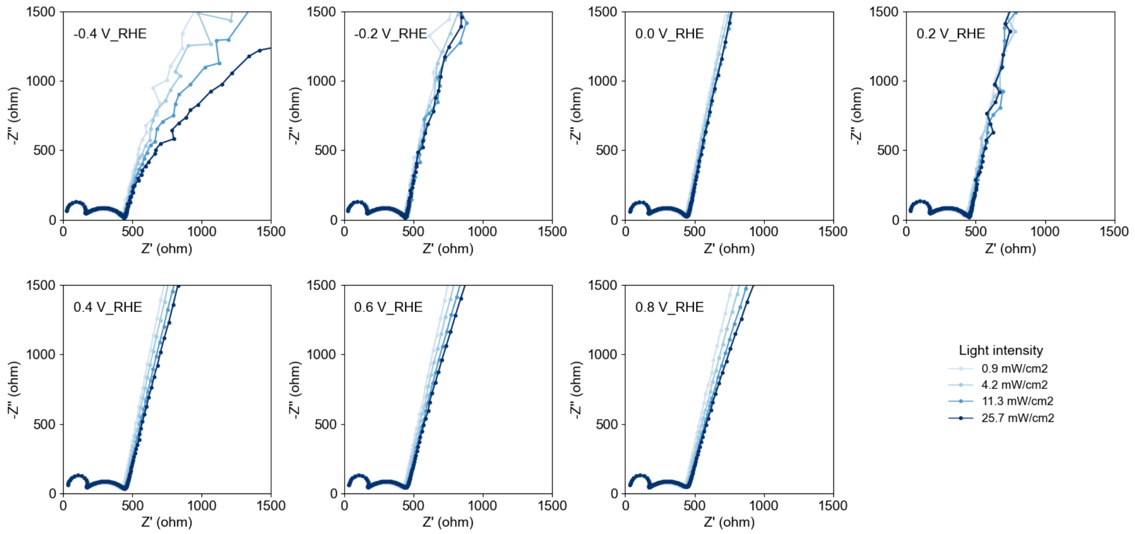

Figure S14. Enlarged view of Figure S13.

In **Figure S12** and **S14**, light-intensity- and applied-potential-independent processes are observed. Based on this behavior, we assign these features not to a photocatalytic process but to a process associated with the back contact between the sample and the FTO substrate. The smaller resistance observed for the 5 mol% Al-doped sample may contribute slightly to its overall performance, although the absolute |Z| value is small. This resistance is not relevant in the slurry-type system, where no substrate contact exists.

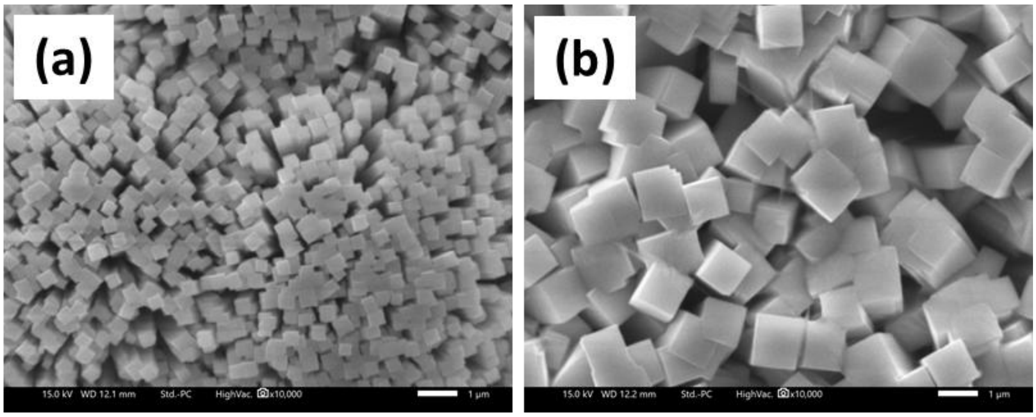

Figure S15. SEM images of (a) Y-doped Sn_3O_4 and (b) Al-doped Sn_3O_4 films.
